# Microbiota–gut–brain axis and its therapeutic applications in neurodegenerative diseases

**DOI:** 10.1038/s41392-024-01743-1

**Published:** 2024-02-16

**Authors:** Jian Sheng Loh, Wen Qi Mak, Li Kar Stella Tan, Chu Xin Ng, Hong Hao Chan, Shiau Hueh Yeow, Jhi Biau Foo, Yong Sze Ong, Chee Wun How, Kooi Yeong Khaw

**Affiliations:** 1 School of Pharmacy, Monash University Malaysia, Jalan Lagoon Selatan, 47500 Bandar Sunway, Selangor Malaysia; 2 School of Pharmacy, Faculty of Health & Medical Sciences, Taylor’s University, 1, Jalan Taylors, Subang Jaya, 47500 Selangor Malaysia; 3 Digital Health & Medical Advancements, Taylor’s University, 1, Jalan Taylors, Subang Jaya, 47500 Selangor Malaysia; 4 School of Biosciences, Faculty of Health & Medical Sciences, Taylor’s University, 1, Jalan Taylors, Subang Jaya, 47500 Selangor Malaysia; 5 UCL School of Pharmacy, University College London, 29-39 Brunswick Square, London, WC1N 1AX UK

**Keywords:** Diseases of the nervous system, Microbiology

## Abstract

The human gastrointestinal tract is populated with a diverse microbial community. The vast genetic and metabolic potential of the gut microbiome underpins its ubiquity in nearly every aspect of human biology, including health maintenance, development, aging, and disease. The advent of new sequencing technologies and culture-independent methods has allowed researchers to move beyond correlative studies toward mechanistic explorations to shed light on microbiome–host interactions. Evidence has unveiled the bidirectional communication between the gut microbiome and the central nervous system, referred to as the “microbiota–gut–brain axis”. The microbiota–gut–brain axis represents an important regulator of glial functions, making it an actionable target to ameliorate the development and progression of neurodegenerative diseases. In this review, we discuss the mechanisms of the microbiota–gut–brain axis in neurodegenerative diseases. As the gut microbiome provides essential cues to microglia, astrocytes, and oligodendrocytes, we examine the communications between gut microbiota and these glial cells during healthy states and neurodegenerative diseases. Subsequently, we discuss the mechanisms of the microbiota–gut–brain axis in neurodegenerative diseases using a metabolite-centric approach, while also examining the role of gut microbiota-related neurotransmitters and gut hormones. Next, we examine the potential of targeting the intestinal barrier, blood–brain barrier, meninges, and peripheral immune system to counteract glial dysfunction in neurodegeneration. Finally, we conclude by assessing the pre-clinical and clinical evidence of probiotics, prebiotics, and fecal microbiota transplantation in neurodegenerative diseases. A thorough comprehension of the microbiota–gut–brain axis will foster the development of effective therapeutic interventions for the management of neurodegenerative diseases.

## Introduction

Microbes have always been an essential part of human life. The co-evolution between the human host and microbes has established a mutualistic symbiosis in which the host provides a hospitable environment and nutrients for the microbiota, while the microbiota exerts substantial influence on the host during homeostasis and disease.^1^ The human gastrointestinal (GI) tract is populated with the most diverse microbial community in the human body, including bacteria, fungi, viruses, and archaea.^2,3^ Approximately 2000 bacterial species have been identified in the human gut, and it is estimated that the gut microbiota contains nearly 150 times more genes than the human genome.^4,5^ The vast genetic and metabolic potential of the gut microbiome underpins its ubiquity in nearly every aspect of human biology, including health maintenance, development, aging, and disease.^6^

The biological importance of the gut microbiome is evident from the early stages of life. The human gut microbiota develops after birth and contributes to the development of the immune system in newborns.^7,8^ Furthermore, microbial colonization in the GI tract of infants enables the production of essential amino acids and vitamins, which begins around 4 months of life.^9^ The gut microbiome gradually reaches an adult-like configuration by the age of 3–6 years old and remains stable throughout adulthood.^10–12^ Notable biological functions of the adult gut microbiome include regulation of nutrient harvest from the diet,^13^ regulation of immunity and auto-immunity,^3,14^ maintenance of intestinal barrier integrity,^15,16^ cholesterol metabolism,^17–19^ transformation of bile acids (BAs),^20,21^ production of antimicrobial peptides,^22,23^ and drug metabolism.^24,25^ Recent studies have revealed that the human gut microbiome is a major determinant of plasma metabolome, potentially playing a more dominant role than genetics.^26–28^ Notably, dysbiosis has been recognized as one of the 12 updated hallmarks of aging, further emphasizing the importance of the microbiome.^29^

**Fig. 1:**
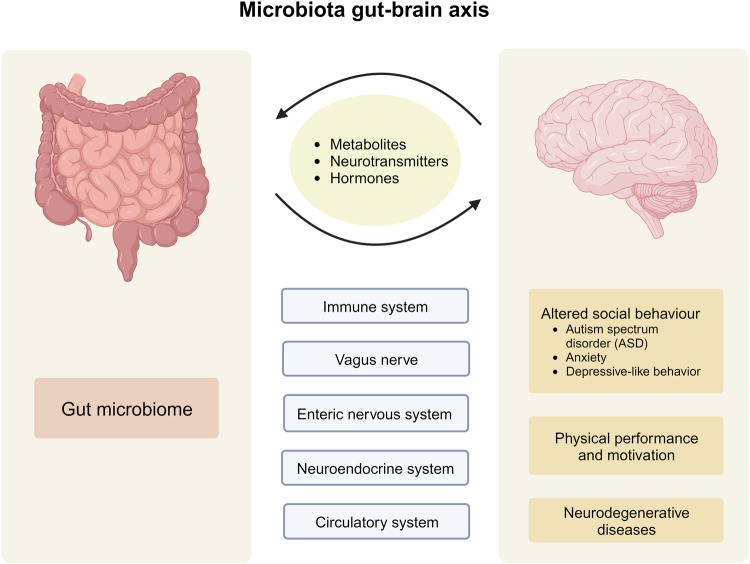
The microbiota–gut–brain axis. The bidirectional communication between the gut microbiome and the brain is mediated by the immune system, vagus nerve, enteric nervous system, neuroendocrine system, and circulatory system. Alterations in gut microbiota have been linked to the development of autism spectrum disorders, anxiety, depressive-like behavior, impaired physical performance, and motivation, as well as neurodegenerative diseases. This figure was created with BioRender (https://biorender.com/)

Accumulating evidence has unveiled the bidirectional communication between the gut microbiome and central nervous system (CNS), referred to as the “microbiota–gut–brain axis”.^30,31^ Although the gut and brain are anatomically separated, several pathways by which the gut microbiota communicates with the CNS have been proposed. These include modulation of the immune system, vagus nerve, enteric nervous system (ENS), neuroendocrine system, and circulatory system via the production of neuroactive substances, metabolites, and hormones (Fig. 1).^31,32^ Studies have shown that gut microbiota is capable of producing or stimulating the production of neurotransmitters, including serotonin,^33–35^ dopamine,^36^ and γ-aminobutyric acid (GABA).^37^ Earlier studies reporting correlations between gut microbiota and CNS functions have largely relied on simplified animal models, which are insufficient to elucidate the underlying mechanisms of action. Nevertheless, the development of new technologies and culture-independent methods has allowed researchers to move beyond correlative studies toward mechanistic exploration to shed light on microbiome–host interactions.^31^ Pre-clinical and human studies have demonstrated the intricate involvement of gut microbiota in the regulation of social behavior,^38–42^ depressive-like behavior,^43–47^ physical performance, and motivation.^48–50^

There is a growing recognition of the role of gut microbiome in neurodegenerative diseases. Notably, early microbiome changes were detected in preclinical Alzheimer’s disease (AD) patients and prodromal Parkinson’s disease (PD) patients.^51–53^ Moreover, studies on animal models have provided compelling evidence that the altered gut microbiome drives neurodegenerative disease pathogenesis, primarily through the modulation of microglial functions and activation.^54–57^ Microglial activation and neuroinflammation are pathological hallmarks of neurodegenerative diseases.^58^ The microbiota–gut–brain axis represents an important regulator of glial functions,^59–62^ making it an actionable target to ameliorate the development and progression of neurodegenerative diseases.

The purpose of this review is to update the current state of knowledge of the mechanisms governing the microbiota–gut–brain axis in neurodegenerative diseases, with a particular emphasis on the interactions between gut microbiome and glial cells (microglia, astrocytes, and oligodendrocytes). We next discuss the roles of gut microbiota-derived metabolites, gut microbiota-related neurotransmitters, and gut hormones in neurodegenerative diseases. While these elements are highly interconnected and interdependent, we present each element separately to enhance clarity and provide focused discussions on their distinct mechanisms and contributions. Subsequently, we examine the potential of targeting the intestinal barrier, blood–brain barrier (BBB), meninges, and peripheral immune system to modulate the microbiota–gut–brain axis and counteract glial dysfunction and neurodegeneration. Finally, we conclude by assessing the pre-clinical and clinical evidence of probiotics, prebiotics, and fecal microbiota transplantation (FMT) in neurodegenerative diseases. In addition, we provide a brief update on the current understanding of the roles of microglia in neurodegenerative diseases.

## Roles of microglia in neurodegenerative diseases

Microglia are the primary innate immune cells of the CNS, accounting for nearly 10% of CNS cells. Although microglia were erroneously considered inert bystanders of CNS disorders, they possessed diverse context-dependent functions central to CNS development, homeostasis, and diseases.^63–65^ Under homeostatic conditions, microglia contribute to the regulation of numerous physiological functions, including neurogenesis,^66,67^ angiogenesis,^68^ maintaining BBB integrity,^69^ synaptic pruning and remodeling,^70,71^ synaptic transmission,^72–74^ myelin health,^75,76^ as well as phagocytosis and removal of apoptotic neurons and cellular debris.^67,77,78^ Microglia actively surveys and responds promptly to various environmental perturbations in the CNS by evoking a broad repertoire of cellular alterations to restore homeostasis.^79,80^

The importance of microglia in AD has been clearly illustrated in a recent spatiotemporal analysis. Among the three major glial cell types (microglia, astrocytes, and oligodendrocytes), microglia are the primary responder to beta-amyloid (Aβ) plaques and accumulate in close vicinity of the plaques (<10 µm).^81^ Several genome-wide association studies (GWAS) have also implicated microglia as the primary cell type expressing AD genes.^82–85^ In addition, growing evidence has implicated microglia in the pathogenesis of PD. Postmortem analysis of ventral midbrains from PD patients revealed a significantly increased number of microglia with an ameboid shape, suggestive of an activated state.^86^ Importantly, studies have identified a significant association between PD risk variants and microglia.^86,87^ However, conflicting results were reported in a single-nuclei transcriptomic atlas of the human substantia nigra (SN), which found no association between PD risk and microglia or astrocytes,^88^ underscoring the imperative for additional comprehensive studies. On the other hand, postmortem transcriptomic analysis of the amyotrophic lateral sclerosis (ALS) spinal cord has reported an increase in inflammatory reactions driven by microglia and astrocytes.^89^ Similarly, the involvement of microglia in frontotemporal dementia (FTD) and Huntington’s disease (HD) is also well documented.^90–92^

A core function of microglia is the efficient recognition and phagocytic clearance of protein aggregates and cellular debris without damaging surrounding tissue to maintain CNS homeostasis.^93,94^ The phagocytic activity of microglia is crucial for the removal of Aβ,^94^ tau,^95^ and α-synuclein.^96^ However, microglial phagocytic activity becomes dysfunctional during aging and neurodegenerative diseases, resulting in the gradual accumulation of toxic compounds and cognitive decline.^93,94^ Moreover, overactive microglial phagocytosis of stressed but viable neurons leads to neuronal loss and neurodegeneration.^97^ Several regulators of microglial phagocytosis have been identified, including but not limited to tyrosine kinase-binding protein (TYROBP),^98^ triggering receptor expressed on myeloid cells 2-apolipoprotein E (TREM2-APOE) pathway,^99,100^ spleen tyrosine kinase (SYK),^101,102^ classical complement system,^103^ purinergic system,^104^ sialic acid binding immunoglobin-like lectins (Siglecs) (CD22 and CD33),^105–107^ TAM system,^77^ and mechanosensor Piezo1.^108,109^

### Mechanisms of microglial activation

**Fig. 2:**
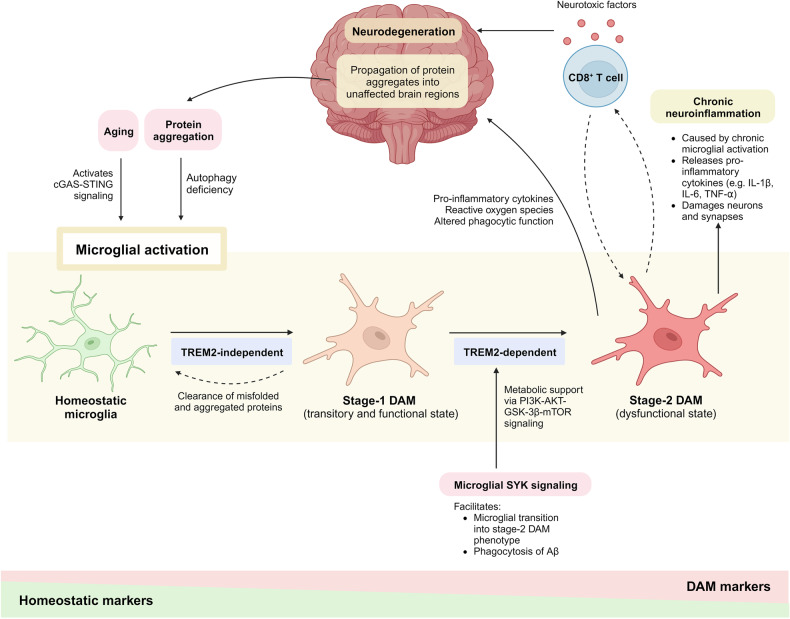
Microglial activation and neurodegeneration. Aging induces microglial activation by activating the cyclic GMP–AMP synthase (cGAS)–stimulator of interferon genes (STING) signaling pathway. Misfolded proteins and protein aggregates induce microglial activation by impairing microglial autophagy. Stage-1 DAM represents a transitory and functional subtype with a higher capacity of phagocytosis initiated by a TREM2-independent mechanism, whereas stage-2 DAM represents a dysfunctional state initiated by a TREM2-dependent mechanism. The microglial spleen tyrosine kinase (SYK) signaling provides metabolic support to facilitate microglial transition into stage-2 DAM. Maladaptive microglial-T-cell signaling drives neurodegeneration by releasing neurotoxic factors. Microglial activation creates a feed-forward vicious cycle that aggravates neurodegeneration as activated microglia contribute to the propagation of protein aggregates into unaffected brain regions. This figure was created with BioRender (https://biorender.com/)

Several genetically distinct subtypes of microglia have been discovered as they respond to signals or challenges in the brain microenvironment, namely homeostatic microglia and “disease-associated microglia” (DAM) or “microglial neurodegenerative phenotype” (MGnD).^99,100^ The DAM was first identified in a 5xFAD mouse model, an amyloid model harboring five mutations associated with familial AD, and was found to cluster in close proximity to the Aβ plaques.^99,110^ The transition from homeostatic state to DAM is associated with the downregulation of homeostatic markers and upregulation of genes related to AD and other neurodegenerative diseases, including APOE, TREM2, and TYROBP. Stage-1 DAM represents a transitory and functional subtype with a higher capacity of phagocytosis initiated by a TREM2-independent mechanism, whereas stage-2 DAM represents a dysfunctional state that contributes to AD pathology initiated by a TREM2-dependent mechanism.^99,111^ This transition also leads to considerable morphological changes, transforming microglia from thin cell bodies with highly ramified extensions into ameba-like cells with fewer branches (Fig. 2).^65^

A recent study on naturally aged mice found that in the absence of an additional trigger, the activation of cyclic GMP–AMP synthase (cGAS)–stimulator of interferon genes (STING) signaling pathway is sufficient to promote aging-related inflammation and neurodegeneration by triggering reactive microglial transcriptional states.^112^ The cGAS-STING signaling pathway is an innate immune sensing system that is capable of driving both acute and chronic low-grade inflammation, which is central to the development of neurodegenerative pathologies.^113^ In addition, intact autophagy is required for effective microglial transition into DAM phenotype and microglial proliferation in response to Aβ plaques in 5xFAD mice. Deletion of autophagy gene *Atg7* led to impaired ability of microglia to engage Aβ plaques and promoted microglial senescence, which was reversed by administration of senolytic drugs.^114^ Nevertheless, the transcriptional states of microglia remain incompletely understood, as microglia may respond to multiple pathological stimuli in the brain simultaneously. For instance, microglia adopt two distinct DAM phenotypes when responding to amyloid pathology and myelin damage in 5xFAD mice with dysfunctional myelin (*Cnp*^*−/−*^5xFAD mice), which may reflect the comorbid state of the aged brain.^115^

More recently, two studies have identified a critical intracellular regulator of microglial activation that acts downstream of TREM2 and CD33 to counteract Aβ pathology, namely SYK.^101,102^ Microglial SYK signaling enables effective microglial response to Aβ by providing metabolic support via the phosphoinositide 3-kinase (PI3K)-AKT-glycogen synthase kinase-3β (GSK-3β)-mammalian target of rapamycin (mTOR) pathway, allowing microglia to acquire complete DAM phenotype. The loss of SYK signaling interfered with microglial clustering around Aβ plaques, microglial transition into complete DAM phenotype, and phagocytosis of Aβ following exposure to Aβ in 5xFAD mice.^101,102^ Conversely, replacing the mutant microglia of *Trem2*^−/−^ 5xFAD mice with *Trem2*^+/+^ circulation-derived myeloid cells through hematopoietic cell transplantation effectively restored microglial activation in response to Aβ plaques. This effect is attributed to the restoration of microglial SYK signaling and the DAM transcriptional program.^116^ The observed favorable outcomes of SYK-mediated complete/stage-2 DAM phenotype in Aβ pathology are in contrast with the prevailing view that stage-2 DAM represents a dysfunctional and pro-inflammatory state. Thus, we recommend caution in oversimplifying DAM states similar to the previous nomenclatures (resting versus activated; M1 versus M2) to account for the inherent plasticity of microglia. More research is needed to elucidate the upstream and downstream mechanisms governing microglial transition across their many states, including a potential exploration of their reversibility.

### Microglia-mediated neuroinflammation

Among the diverse microglial functions, microglia-mediated neuroinflammation has received attention due to its complex and dynamic role in health and disease. Early neuroinflammation is protective as it promotes tissue repair, cellular debris clearance, and pathogen removal.^117^ Furthermore, early neuroinflammation has been shown to be an adaptive mechanism by microglia that protects against AD pathology by reducing the levels of Aβ and tau.^118–120^ Similarly, early microglial activation assists in the clearance of neuronal human TAR DNA-binding protein 43 (hTDP-43) and motor neuron recovery in the ALS mouse model.^121^ However, microglia lose their homeostatic molecular signatures and become progressively activated with increasing age or during pathological conditions, transitioning into distinct disease-associated phenotypes with sustained release of pro-inflammatory cytokines and chemokines.^122–124^ Chronic microglial activation leads to persistent low-grade neuroinflammation that is detrimental to neurons and synapses, leading to neurodegeneration.^125^ Indeed, neuroinflammation and microglial activation are consistent features across neurodegenerative diseases, including AD,^126–128^ PD,^86,129^ HD,^130,131^ FTD,^91,132^ and ALS.^89,133–135^

Numerous studies have reported that misfolded proteins and protein aggregates, including tau,^136–139^ Aβ,^138,140^ α-synuclein,^141–144^ mutant huntingtin,^145^ TDP-43,^132,146^ superoxide dismutase 1 (SOD1),^147–149^ and fused in sarcoma (FUS)^150^ induce microglial activation and neuroinflammation. Autophagy deficiency induced by protein aggregates has been shown to be a major driver of microglial activation (Fig. 2). Prolonged exposure to Aβ impairs microglial autophagy by inducing lysosomal dysfunction, resulting in microglial activation.^151^ Autophagy deficiency disrupts microglial response to Aβ by inhibiting DAM development and inducing microglial senescence.^114^ Moreover, the loss of functional microglial autophagy is deleterious as it exacerbates tau pathology and spreading in PS19 tau transgenic mice,^152^ as well as contributes to elevated release of pro-inflammatory cytokines and NLR family pyrin domain-containing 3 (NLRP3) inflammasome activation in *Becn1*^+/−^ APP/PS1 mice.^153^ Activated microglia also release chemokines that disrupt neuronal autophagy by altering the neuronal C-C chemokine receptor type 5 (CCR5)-mTORC1-autophagy pathway in HD and tauopathy mice.^154^

In PD models, α-synuclein inhibits microglial autophagy by triggering toll-like receptor 4 (TLR4)-dependent p38 mitogen-activated protein kinase (MAPK) phosphorylation and activating the AKT-mTOR signaling cascade.^144,155^ This leads to a self-perpetuating cycle that further exacerbates neuroinflammation in PD as microglia with impaired autophagy have elevated pro-inflammatory responses and lose the ability to clear α-synuclein, resulting in neurodegeneration.^144,156,157^ *C9orf72* mutation, the leading genetic cause of ALS and FTD, disrupts microglial autophagy and triggers sustained activation of NLRP3 inflammasome and nuclear factor-κB (NF-κB) signaling in human-induced pluripotent stem cell-derived microglia-like cells (hiPSC-MG). The dysfunctional microglial autophagy aggravates motor neuron death in microglia-motor neurons co-culture following excitotoxic insult, a key pathomechanism in ALS.^158^ Ultimately, the activation of microglia by these protein aggregates creates a feed-forward vicious cycle that aggravates neurodegeneration as activated microglia contribute to the propagation of tau, Aβ, α-synuclein, and TDP-43 into unaffected brain regions.^159–164^

Recently, the intricate interplay between microglia, tauopathy, APOE, and T cells in driving neurodegeneration has been elucidated.^165^ APOE is a lipid and cholesterol transporter with numerous CNS-related functions, including regulation of microglial and astrocytic functions,^100,166,167^ cerebrovascular integrity,^168^ BBB integrity,^169,170^ myelin dynamics,^166,171^ and neuronal network activity.^172^ APOE exists in three isoforms, namely APOE2, APOE3, and APOE4, among which APOE4 isoform has been identified as the strongest genetic risk factor for late-onset AD.^173^ Studies have demonstrated that APOE4 drives Aβ- and tau-mediated neurodegeneration by inducing microglial and astrocytic activation.^174–179^ In addition, APOE4 genetic background drives an accelerated spread of α-synuclein pathology and neurodegeneration.^180,181^

In light of the strong correlation between tau pathology and brain atrophy in AD, rather than Aβ,^182^ a comparison was made between the immune responses of amyloid-depositing APP/PS1-21 (A/PE4) and 5xFAD (5xE4) mice, versus P301S tau transgenic (TE4) mice expressing human APOE4.^165^ Interestingly, the number of T cells was significantly increased only in TE4 mice in regions where brain atrophy occurred, and this increase was positively correlated with the number of microglia. Further sequencing analysis on the T cells revealed that TE4 mice carried increased activated CD8^+^ T cells and reduced exhausted T cells, suggesting that the T-cell activation drives tau-mediated neurodegeneration. The study also demonstrated that interfering with the immunological hub between activated microglia and T cells using cell-depleting treatments attenuated tau-mediated neurodegeneration. Microglial depletion reduced CD3^+^ and CD8^+^ T cells and attenuated tau pathology in TE4 mice. Conversely, T-cell depletion induced microglial transition from an activated state to a homeostatic state, along with reduced tau pathology in TE4 mice.^165^ These findings are in concordance with a recent study that reported a detrimental synergism between microglia and CD8^+^ T cells in exacerbating neuronal and glial damage.^183^

Similar maladaptive microglial-T-cell signaling also drives neurodegeneration in the α-synuclein-driven PD mouse model, which was ameliorated following genetic knockout or pharmacological depletion of T cells.^184^ Furthermore, infiltration of T cells in the CNS drives microglial and astrocytic activation in two different ALS mouse models (hSOD1^G93A^ and TDP-43^A315T^ mice). These pathological changes were largely prevented by reducing immune cell infiltration using natalizumab, accompanied by reduced motor neuron degeneration, delayed onset of paralysis, and prolonged survival.^185^ Together, these data indicate that microglial-T-cell signaling offers a prospective avenue for tackling neuroinflammation and neurodegeneration.

## Microbiota–gut–brain axis in neurodegenerative diseases

### Interaction between gut microbiota and microglia

The interaction between microglia and gut microbiota begins early in life. A recent study demonstrated that early-life administration of a broad-spectrum antibiotic cocktail led to altered microglial morphology and myelin-related gene expression in adolescent mice, accompanied by anxiety-like and compulsive-like behaviors.^186^ Throughout the host lifespan, the gut microbiome provides essential signals to microglia during health and disease.^59,60,187,188^ Notably, among the neuronal and glial cells, microglia are the most vulnerable to alterations in the gut microbiome.^189^

Under homeostatic conditions, the gut microbiome is responsible for regulating microglial maturation and activation via short-chain fatty acids (SCFAs) release.^190,191^ Erny and colleagues found that germ-free (GF) mice and antibiotic-treated mice suffered from impaired microglial immune responses when challenged with lipopolysaccharide (LPS) and lymphocytic choriomeningitis virus (LCMV) infection. However, the microglial defects and immaturity were partially restored by recolonization with complex microbiota and SCFAs supplementation.^191^ In a subsequent study, Erny et al. discovered that the host microbiota regulates microglia mitochondrial functions and identified acetate as the major SCFA-rescuing microglial homeostasis in GF mice.^190^ In addition, the gut microbiota plays a role in facilitating the transition of microglia to DAM phenotype during aging, as specific-pathogen-free (SPF) aged mice display higher expression of DAM-related genes than GF-aged mice.^192^ Antibiotic-induced gut microbiota depletion stimulates global reduction of Ly6C^hi^ monocytes pool and promotes Ly6C^hi^ monocytes transition towards a pro-inflammatory state. The elevated immune activation is coupled with microglial activation, impaired hippocampal synaptic transmission, and cholinergic gamma oscillations.^193^ Additional evidence suggests that reshaping the gut microbiome of high-fat diet (HFD)-fed obese mice with dietary fibers successfully mitigated the cognitive and social impairments of their offspring by alleviating the microglial maturation defects. SCFAs supplementation in the offspring with acetate and propionate promoted microglial maturation and reduced maternal obesity-induced cognitive and social deficits.^194^

Aside from the regulation of microglial homeostasis, gut microbiota-derived metabolites also play a crucial role in triggering microglial cell death.^195^ During aging, the increased level of gut microbial metabolite isoamylamine (IAA) crosses the BBB and induces microglial apoptosis by activating the S100 calcium-binding protein A8 (S100A8) signaling. Specifically, an increased abundance of *Ruminococcaceae* and reduced *Ruminococcaceae*-targeting bacteriophage family *Myoviridae* were observed in the gut of aged mice and elderly people, contributing to increased IAA. IAA binds to the promoter region of S100A8 and interrupts its hairpin structure, facilitating p53 access to the S100A8 promoter region. The study further demonstrated that IAA administration induced cognitive decline in young mice, whereas IAA reduction attenuated the neuronal loss and cognitive deficits of aged mice.^195^

In this section, we present evidence of the interaction between gut microbiota and microglia in different neurodegenerative diseases.

#### Alzheimer’s disease

Accumulating evidence has demonstrated the interaction between gut microbiota and microglia in AD. In the triple transgenic AD (3xTg-AD) mouse model, the development of AD pathologies, including Aβ plaque, hyperphosphorylated tau, synaptic dysfunction, and microglial activation appears to be influenced by the gut microbiome. This is evident as SPF 3xTg-AD mice exhibit greater AD pathologies compared to GF 3xTg-AD mice. Importantly, FMT from AD patients to GF 3xTg-AD mice restored the main AD pathologies and microglial activation.^196^

Similar findings have been reported in GF and antibiotic-treated amyloidogenic APP/PS1 mice.^54,197–199^ It was reported that GF condition confers protection against Aβ pathology and microglial activation in *APP*_*SWE*_*/PS1*_*L166P*_ (APP/PS1-21) mice. However, this protection was diminished following FMT from 12-month-old conventionally raised APP/PS1-21 mice to 4-month-old GF APP/PS1-21 mice.^198^ Similar trends were also observed in APP/PS1-21 following gut microbiota depletion using long-term (5-week) and short-term (7-day) antibiotic treatment.^54,197^ Long-term perturbation of gut microbiome using antibiotic cocktail resulted in reduced Aβ deposition, reduced plaque-localized microglia and altered transcriptional profile of microglia (increased homeostatic microglial genes and decreased MGnD genes) in 7-week-old male APP/PS1-21 mice. Interestingly, these effects were absent in female mice, suggesting potential sexual dimorphism in their responses to gut microbiome manipulation. Importantly, the AD pathologies in antibiotic-treated male mice were partially restored after 3-week FMT from age-matched male APP/PS1-21 mice.^197^

Recently, growing studies have illustrated the presence of critical windows of microbial development, during which early-life modulation of the gut microbiome has a long-lasting impact on different aspects of physiology.^186,200–202^ To further validate the time-specific role of the gut microbiome in AD, Dodiya et al. repeated the experiment using short-term 7-day antibiotic treatment administered from postnatal day 14 to day 21, and sacrificed the mice at 9 weeks of age. Consistent with their previous findings,^197^ short-term antibiotic treatments effectively reduced Aβ pathology, reduced DAM population, and expression of microglial sensome genes in male APP/PS1-21 mice, which were reversed by FMT from age-matched mice. Interestingly, microglia depletion using colony-stimulating factor 1 receptor inhibitor, PLX5622, mitigated the protective effect of antibiotic treatment against amyloidosis. These results suggest that microglia are essential mediators of the microbiota–gut–brain axis in Aβ pathology.^54^

Similarly, the gut microbiota is required for microglial activation in 5xFAD mice.^203–205^ 5xFAD mouse model expresses five familial AD mutations and develops Aβ accumulation and gliosis as early as 2 months of age, synaptic degeneration by 4 months of age, and cognitive impairment as early as 4–5 months of age.^206,207^ It was found that gut microbiota ablation using 5-month antibiotics treatment prevented microglial activation in 7-month-old 5xFAD mice by reducing immune cell infiltration.^203^ Moreover, 5-month antibiotics treatment alleviated AD pathologies and microglial activation in 6.5-month-old 5xFAD mice by inhibiting CCAAT/enhancer binding protein β/asparagine endopeptidase (C/EBPβ/AEP) signaling.^204^

The involvement of the gut microbiome in facilitating microglial activation is also evident in tau-mediated neurodegeneration. A recent study demonstrated the interplay between gut microbiota, tau and APOE in AD.^56^ Seo and colleagues genetically engineered P301S tau transgenic mice to express different isoforms of human APOE (APOE3 and APOE4) and raised them in conventional or GF environments. Compared to conventionally raised P301S mice expressing human APOE4 (TE4 mice), their GF counterparts showed reduced signs of neurodegeneration (brain atrophy) and neuroinflammation (microglial and astrocytic activation). However, FMT from sex-matched conventionally raised TE4 mice mitigated the neuroprotective effects of GF conditions, indicating that the gut microbiota is responsible for the emergence of tau-mediated neurodegeneration. To further investigate the role of gut microbiota, the researchers induced early-life gut microbiota perturbation through a short-term antibiotic treatment administered from postnatal day 16 to day 22, and sacrificed the mice at 40 weeks of age. Interestingly, they observed sex-dependent and APOE isoform-dependent neuroprotection as the neuroprotective effects of antibiotic treatment were more pronounced in male mice expressing human APOE3 (TE3 mice).^56^ The sex-dependent neuroprotective effects of microbiome perturbations in tau pathology are reminiscent of that observed in amyloid pathology,^197^ underscoring the need for future research to consider the gender effects.

Aging is the predominant risk factor for neurodegenerative diseases as a result of the lifetime accumulation of neuropathologies.^208^ Notably, the gut microbiome of centenarians is associated with “youth-like” signatures (depletion of inflammatory pathobionts and enrichment of beneficial commensals), showing high similarity to those of young individuals.^209^ To investigate whether the acquisition of “youth-like” microbiota could restore aging-induced neurocognitive and immune impairments, Boehme et al. compared the effects of FMT from young (3–4 months; yFMT) or old (19–20 months; oFMT) mice into old recipient mice. Hippocampal transcriptomic analysis revealed that yFMT reversed the aging-induced alterations in the expression of six microglial sensome genes in old mice. These genes included *Trem2*, *Dap12*, *C1qb*, *Fcgr2b*, *Gpr84*, and *Tlr13*.^210^ Of note, DAP12, also known as TYROBP, is an immunoreceptor tyrosine-based activation motifs (ITAM)-containing transmembrane adapter that associates with TREM2.^98^ *Dap12*, along with *Apoe*, were among the most robustly upregulated genes during the microglial transition from homeostatic state to DAM phenotype.^99^ On the other hand, complement component 1q (C1q) is the initiating protein of the classical complement pathway predominantly produced by microglia in the brain.^211^ Studies on P301S tau transgenic mice and plaque-bearing mouse models have shown that C1q binds to synapses and facilitates microglial phagocytosis of synapses.^103,212^ Recent evidence also revealed that the complement-dependent synapse elimination in P301S mice involves coordinated action between microglia and astrocytes.^213^

Altogether, the evidence from different mouse models consistently underscores the significance of the microbiota–gut–brain axis in AD pathologies.

#### Parkinson’s disease

Although GI symptoms and gut microbiota alterations are common in PD patients during the disease course,^214,215^ the underlying mechanisms linking the gut microbiome and PD have only been unveiled recently. The first corroboration arises from the study by Sampson et al., which demonstrated that the development of α-synuclein pathology, microglial activation, and motor deficits in α-synuclein-overexpressing (ASO) mice appear to be influenced by the gut microbiome. This is evident as SPF ASO mice exhibit greater PD pathologies compared to their GF and antibiotic-treated counterparts. Importantly, FMT from PD patients to GF ASO mice restored the main disease features, including α-synuclein-mediated motor dysfunction.^55^ In another study, transgenic rats overexpressing α-synuclein displayed progressive gut dysbiosis with aging, whereas a short-term antibiotic treatment mitigated α-synuclein expression in the forebrain.^216^ Furthermore, FMT from 1-methyl-4-phenyl-1,2,3,6-tetrahydropyridine (MPTP)-treated mice induced motor impairments and neurotransmitter loss in healthy mice. Conversely, FMT from healthy mice ameliorated gut dysbiosis and PD pathologies in MPTP-induced mice, including gut inflammation, glial activation, neurotransmitter abnormalities, and motor dysfunction.^217^ In addition, the development of GI dysfunction and motor symptoms following chronic rotenone administration occurs only in conventionally raised mice, but not in GF mice.^218^ These studies substantiated the significance of the microbiota–gut–brain axis in the pathogenesis of PD.

#### Amyotrophic lateral sclerosis

The motor neuron injury in ALS is thought to arise from multiple interacting pathophysiological mechanisms, including glial dysfunction and neuroinflammation.^89,219,220^ Compared to AD and PD, the roles of gut microbiota in ALS remain relatively understudied. Nevertheless, growing evidence has indicated alterations in the gut microbiome and impaired intestinal barrier integrity in both ALS patients and animal models.^221–225^

Notably, a gradual reduction in the abundance of *Akkermansia muciniphila* and reduced bacterial production of nicotinamide (NAM) were observed during the course of disease in SOD1^G93A^ mice. Conversely, supplementation of *A. muciniphila* was associated with increased motor neurons in the spinal cord, improved motor function, reduced brain atrophy, and prolonged lifespan.^223^ The gut microbiome is also closely linked to *C9orf72* function.^57^ The hexanucleotide (GGGGCC) repeat expansion in the *C9orf72* gene has been identified as the leading genetic cause of ALS and FTD.^226–228^ It was found that the gut microbiota is a potent modifier of disease severity and microglial activation in *C9orf72*-mutant mice raised in different environments. Specifically, *C9orf72*^−/−^ mice reared at pro-inflammatory environment (Harvard Institute) exhibit significantly different gut microbiota composition, greater autoimmune and inflammatory phenotypes, microglial activation, and shorter lifespan as compared to the *C9orf72*^−/−^ mice reared at pro-survival environment (Broad Institute). Importantly, FMT from *C9orf72(Broad)*^−/−^ mice significantly ameliorated the autoimmune and inflammatory phenotypes in *C9orf72(Harvard)*^−/−^ mice.^57^

Alterations in the gut microbiome have been observed in SOD1^G93A^ mice prior to the onset of motor dysfunction, muscle atrophy, and immune cell activation in the spinal cord.^224^ In addition, gut dysbiosis precedes the aggregation of human-SOD1^G93A^ protein in the colon and intestine, along with the development of ENS dysfunction in SOD1^G93A^ mice.^222^ These studies indicate an early microbial contribution to ALS disease pathology.

Interestingly, conflicting results have been reported regarding the consequences of gut microbiota depletion in ALS mouse models. Studies have demonstrated the beneficial effects of antibiotic treatment in ameliorating ALS pathologies. Antibiotic treatment has been shown to inhibit SOD1^G93A^ aggregation in the intestines of SOD1^G93A^ mice, coupled with improved enteric neuromuscular function.^222^ Similar beneficial effects were observed in *C9orf72*-mutant mice. The administration of antibiotics, either prior to the onset or after the establishment of inflammation, effectively suppressed the emergence of inflammatory and autoimmune phenotypes in *C9orf72*^−/−^ mice. Moreover, lifelong antibiotics administration prevented the accumulation of infiltrating myeloid cells within the spinal cord and microglial activation.^57^

However, detrimental effects of antibiotic treatment have also been reported in SOD1^G93A^ mice. Long-term antibiotic treatment induces motor neuron death in the spinal cord and brain atrophy, thereby exacerbating motor abnormalities in SOD1^G93A^ mice.^223^ In addition, antibiotic-induced dysbiosis markedly worsened disease progression in SOD1^G93A^ mice by downregulating homeostatic microglial genes and upregulating MGnD signatures in the spinal cord.^229^ Presently, there is a scarcity of research utilizing GF SOD1 mice due to the considerable challenge of rederivation, which is associated with high mortality rates.^223^ Further investigations are warranted to understand the roles of the gut microbiome and the implications of gut microbiota depletion in ALS. With the growing characterization of ALS-associated genes and the development of ALS animal models (extensively reviewed in ref. ^230^), we recommend future studies to move beyond SOD1 mice. This is relevant considering that SOD1-ALS accounts for only about 12% of familial and less than 2% of sporadic ALS cases.^231^

#### Huntington’s disease

HD is an autosomal dominant neurodegenerative disease caused by a CAG repeat expansion in the huntingtin (*HTT*) gene. This results in misfolding and accumulation of mutant huntingtin protein in brain cells, including neurons, microglia, and astrocytes.^232^ In addition to motor, cognitive, and psychiatric abnormalities, HD patients experience a range of GI disturbances, including nutrient deficiency, diarrhea, and unintended weight loss.^233,234^ However, it was only recently that gut dysbiosis has been revealed in preclinical HD models and HD patients, and studies examining the interaction between gut microbiota and microglia remain absent.

The initial evidence of gut dysbiosis emerged from the R6/1 transgenic mouse model of HD. A notable difference in gut microbiota composition was observed between R6/1 mice and wild-type (WT) mice at 12 weeks of age (early disease stage), which coincided with the manifestation of motor deficits and weight loss.^235^ Gut dysbiosis and intestinal barrier impairment were also detected in R6/2 mice. It was found that R6/2 mice exhibited increased intestinal permeability and reduced colon length at 16 weeks of age (early-mid disease stage), as compared to age-matched WT mice.^236^ Moreover, a higher relative abundance of Bacteroidetes and a lower relative abundance of Firmicutes were reported in both R6/1 and R6/2 mice, as compared to WT mice.^235,236^ However, a notable limitation of the current studies is the lack of metabolomic analyses, which greatly hinders our understanding of the metabolites that regulate HD pathogenesis.

A recent study on HD gene expansion carriers (HDGECs) has identified an altered gut microbiome compared to age-matched and gender-matched healthy controls. Moreover, a reduced abundance of *Eubacterium hallii* was associated with increased severity of motor deficits.^237^ *E. hallii* is a major butyrate-producing species with important health implications, and its depletion has been linked to several diseases.^238–240^ In addition, *E. hallii* has also been shown to influence BA metabolism.^241,242^ However, the study did not observe an increase in Bacteroidetes and a reduction in Firmicutes, as reported in both R6/1 and R6/2 mice.^235,236^

Interestingly, the degree of gut dysbiosis appears to be influenced by the gender of the mice. This difference is evident in male R6/1 mice, which develops greater gut microbiota alterations than female R6/1 mice at 8 weeks of age. Moreover, the plasma levels of acetate were elevated only in male R6/1 mice at 14 weeks of age.^243^ Similar sexual dimorphism was observed in the application of FMT. A recent study performed FMT from WT mice into R6/1 mice and found that male R6/1 mice exhibited greater resistance to FMT engraftment when compared to female R6/1 mice. Consequently, the cognitive function of male R6/1 mice showed no discernible improvement as compared to their female counterparts. However, FMT is ineffective in ameliorating gut dysfunction and motor functions of R6/1 mice.^244^

### Interaction between gut microbiota and astrocytes

Astrocytes are the most abundant glial cells in the CNS with an expanding repertoire of functions, making them a subject of growing research interest. Astrocytes are integral to the maintenance of CNS homeostasis, and any disruptions in their functions contribute to the development of neuropathologies. Importantly, growing studies have revealed the bidirectional signaling between astrocytes and microglia in driving neuroinflammation and neurodegeneration.^245,246^

Emerging evidence has elucidated the communication between gut microbiota and astrocytes across health and disease. For instance, the gut microbiota metabolizes tryptophan into various indole derivatives that act as ligands for the aryl hydrocarbon receptor (AHR) expressed within astrocytes and microglia. The AHR activation suppresses NF-κB signaling and inhibits CNS inflammation in experimental autoimmune encephalomyelitis (EAE) mouse models of multiple sclerosis.^247,248^ In addition, the administration of indole-3-propionic acid attenuated the activity of neurotoxic reactive A1 astrocytes in a mouse model of ischemic stroke.^249^ Furthermore, the gut microbiota is involved in restricting neuroinflammation by promoting anti-inflammatory tumor necrosis factor-related apoptosis-inducing ligand-positive (TRAIL^+^) astrocytes and inducing T-cell apoptosis via TRAIL-death receptor 5 (DR5) signaling.^250^

A recent in vitro study has found that butyrate stimulated adenosine triphosphate release from astrocytes in a cytosolic Ca^2+^-dependent manner, suggesting a potential neuroprotective mechanism worth further exploration.^251^ The astrocytic calcium signaling underlies vital physiological functions, and its dysregulation has been associated with neuroinflammation and neurodegeneration.^246,252^ Gut microbiota manipulation has also been linked to alterations in astrocytic proliferation and functions.^253–256^ However, these studies are limited by the lack of tools to characterize the astrocytes and largely rely on glial fibrillary acidic protein (GFAP) densitometry, which provides limited insights and characterization of astrocytes. It was recommended to be cautious in interpreting the increased number of GFAP^+^ cells as an increase in reactive astrocytes, as GFAP content alone is not a definitive indicator of their reactivity or altered functions.^257^ For a comprehensive overview of the improved tools, approaches, and potential markers to unravel astrocyte biology, we recommend referring to the excellent reviews by Escartin et al.^257^ and Yu et al.^258^

#### Alzheimer’s disease

The effects of gut microbiota manipulation on astrocytes in AD have only emerged recently. Gut microbiota perturbation has also been shown to reduce reactive astrogliosis, promote a shift in astrocytes towards a more homeostatic-like state, and protect against amyloidosis and tau-mediated neurodegeneration. Interestingly, these effects appear to be more prominent in male mice.^56,61^ The sexual dimorphism in astrocytic responses to gut microbiome perturbation is reminiscent of that in microglia, further underscoring the importance of future research to account for gender effects. However, the neuroprotective effects of gut microbiota depletion were diminished following microbiota restoration and SCFA supplementation. In particular, FMT from age-matched control mice restored astrogliosis in antibiotic-treated APP/PS1-21 mice, while SCFAs supplementation restored the gliosis and tau pathology in GF TE4 mice.^56,61^ Although preliminary, these findings in animals demonstrated the involvement of gut microbiome in facilitating the development and progression of AD pathologies, including the modulation of astrocytic responses.

#### Parkinson’s disease

Our understanding of the roles of astrocytic dysfunction and pro-inflammatory glial responses in PD pathogenesis is expanding.^246^ The amelioration of gut dysbiosis in MPTP-induced mice via FMT, dietary intervention, and probiotic administration has been shown to alleviate neuroinflammation and dopaminergic neuron loss. These effects are attributed to the reduction of glial activation, as well as the restoration of metabolite and neurotransmitter abnormalities.^217,259–261^ In addition, it was found that FMT from healthy human controls mitigated MPTP-induced dysbiosis and neurotoxicity in MPTP-induced mice, whereas FMT from PD patients exacerbated these pathologies.^261^ Taken together, these findings corroborate the contributory roles of gut microbiota in driving glial activation and neuropathologies.

### Interaction between gut microbiota and oligodendrocytes

Oligodendrocytes are myelin-forming glial cells in the CNS that myelinate axons to facilitate axonal conduction and provide metabolic support to axons. The axon-supporting functions of oligodendrocytes are critical for the maintenance of motor, sensory, and cognitive functions.^262,263^ Although oligodendrocyte pathology is extensively explored in demyelinating disorders, accumulating evidence suggests its involvement in the pathogenesis of neurodegenerative diseases, including AD,^110^ PD,^86^ ALS,^89^ and HD.^264^

Studies elucidating the interaction between gut microbiome and oligodendrocytes have only emerged recently. Notably, the gut microbiome has been shown to modulate oligodendrocyte maturation and myelin production. Perturbations in gut microbiome, including GF conditions and antibiotic treatment, have been shown to trigger excessive myelination in the prefrontal cortex by inducing oligodendrocyte maturation and upregulating myelin-related genes.^62,265^ Importantly, these alterations in GF mice were ameliorated by colonization with a conventional microbiota following weaning.^265^ Moreover, the administration of tributyrin, a prodrug of butyrate, rescued the myelin dysregulation and behavioral deficits in antibiotic-treated mice.^62^ The beneficial effects of butyrate on oligodendrocytes are similarly evident in cuprizone- and lysolecithin-induced demyelination.^266^ Furthermore, the gut microbiota facilitates the conversion of dietary tyrosine to 4-ethylphenol (4EP), which is further sulfated into 4-ethylphenyl sulfate that can enter the brain. Colonization of GF mice with 4EP-producing bacteria leads to reduced oligodendrocyte maturation and neuronal myelination, ultimately promoting anxiety-like behavior and altered social communication.^39^ Nevertheless, the specific impact of these modulations on oligodendrocyte functions within the context of other neurodegenerative diseases remains to be clarified. Given the growing recognition of the microglia-oligodendrocyte interplay and astrocyte-oligodendrocyte interplay in regulating myelin health,^76,267^ we anticipate that the gut microbiome may exerts its influence on oligodendrocytes by modulating microglia and astrocytes, thus suggesting the need for further investigation.

Emphasis should also be placed on oligodendrocyte precursor cells (OPCs). Aside from their canonical role in maturing into myelinating oligodendrocytes, recent evidence suggests that OPCs are also involved in the regulation of proper guidance of interneurons, axonal regeneration, angiogenesis, and inflammatory processes.^268^ The administration of antibiotics has been shown to impair OPC differentiation following lysolecithin-induced demyelination, resulting in fewer differentiated oligodendrocytes within the demyelinated lesions. However, the OPC differentiation and extent of remyelination were unaltered in cuprizone-treated GF mice when compared to their SPF counterparts.^269^ These findings underscore the complexity of microbiome perturbation on OPCs and the need for further research, including their relevance within the context of other neurodegenerative diseases.

## Gut microbiota-derived metabolites in neurodegenerative diseases

The gut microbiota contributes to host physiology and brain health by generating a variety of metabolites through bacterial de novo metabolism and by modifying host-derived molecules.^31,270^ In this review, we discuss the mechanisms of the microbiota–gut–brain axis in neurodegenerative diseases using a metabolite-centric approach. The presence of a species possessing specific biosynthetic capabilities does not guarantee in vivo production of downstream metabolites in pharmacologically relevant quantities. Moreover, multiple gut microbes can produce the same metabolite. Thus, examining the gut microbiota through a functional metabolic lens (metabolite-centric), rather than focusing on taxonomic or phylogenetic aspects, is more valuable for understanding the intricate interactions between the microbiota and the host.^271,272^

### Short-chain fatty acids mitigate neuroinflammation and neurodegeneration

The microbial fermentative activity of gut microbiota is vital for the production of SCFAs, including butyrate, acetate, and propionate, from non-digestible dietary fibers.^271,273^ SCFAs are saturated fatty acids composed of one to six carbon atoms. The predominant SCFAs found in the human body are acetate (C2), propionate (C3), and butyrate (C4), which comprise ~95% of the total SCFA pool.^271^ Numerous studies have illustrated the link between SCFAs and human physiological processes, including immunity,^274,275^ intestinal homeostasis,^276–278^ cholesterol metabolism,^17^ and control of glucose homeostasis and energy balance.^279–281^

SCFAs exert their physiological activities by acting as endogenous ligands for G-protein-coupled receptors (GPCRs), and modulating gene expression by inhibiting histone deacetylases (HDACs).^282^ GPCRs are the largest family of cell surface receptor proteins that regulate diverse physiological and pathological processes, and as such, are one of the most intensively studied targets for drug development.^283^ Moreover, GPCRs play a pivotal role in enabling the nervous system to accurately respond to external stimuli and internal states.^284^ SCFAs are endogenous ligands for a subset of GPCRs, including GPR43 and GPR41, which were subsequently renamed as free fatty acid receptor 2 (FFAR2) and FFAR3, respectively. Another important GPCR activated by SCFA is GPR109A, also known as hydroxycarboxylic acid receptor 2 (HCAR2), which is activated by butyrate and β-D-hydroxy butyrate.^282^ It was reported that the FFAR2-deficient SPF mice developed microglial defects resembling GF mice.^191^ In addition, an in vitro study has demonstrated that acetate exerts anti-inflammatory effects in Aβ-induced BV-2 microglial cells by upregulating the levels of GPR41 and inhibiting the ERK/JNK/NF-κB signaling pathway.^285^

On the other hand, HDACs are part of the epigenetic regulatory mechanisms that control gene expression. Histone deacetylation by HDACs is associated with transcriptional repression by inducing a closed chromatin structure. Dysregulated epigenetic regulations and the consequent impact on gene expression and cellular processes are important contributors to aging and age-related human pathologies, including neurodegenerative diseases.^286,287^ Among the SCFAs, butyrate is the most potent HDAC inhibitor that is generally thought to inhibit the activity of class I HDACs (HDAC1, −2, −3, and −8) and class IIa HDACs (HDAC4, −5, −7, and −9), but not class IIb HDACs (HDAC6 and HDAC10) and class III HDACs (sirtuins).^288^ Acetate and butyrate have been shown to inhibit the inflammatory response of LPS-stimulated primary microglia by inhibiting HDAC activity and NF-κB activation.^289^ Furthermore, the inhibition of microglial HDAC1 expression by propionate and butyrate has been shown to alleviate microglial activation and reduce the levels of pro-inflammatory factors in GF mice.^290^ Conversely, the anti-inflammatory effects of butyrate on LPS-induced BV-2 cells were blocked by HDAC3 agonist ITSA-1 and MCT1 inhibitor AZD3965.^291^

#### Alzheimer’s disease

Several studies have reported reduced SCFAs-producing species and SCFA levels in individuals with mild cognitive impairment (MCI) and AD patients.^292–295^ Notably, reduced fecal levels of SCFAs were negatively associated with Aβ deposition in patients with MCI.^293^ In addition, increased levels of HDAC2 and HDAC6 were detected in AD mouse models and AD patients.^296,297^ Thus, HDAC inhibition represents a promising approach for the treatment of AD. This is exemplified by the notable findings that the genetic deletion of microglial *Hdac1* and *Hdac2* substantially ameliorated the cognitive deficits of 5xFAD mice by enhancing microglial phagocytosis of Aβ.^298^ Despite numerous studies supporting the pivotal roles of SCFAs in mediating gut microbiota-microglia communication, mechanistic studies elucidating the underlying mechanisms of SCFAs in AD remain limited and yield conflicting results.

**Fig. 3:**
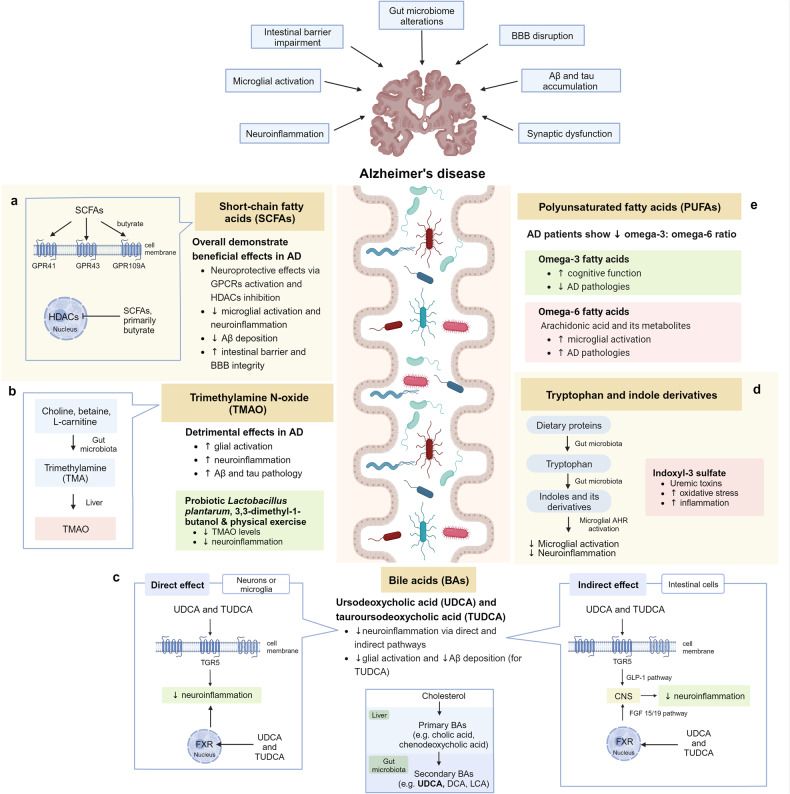
Microbiota–gut–brain axis in Alzheimer’s disease. **a** Short-chain fatty acids (SCFAs) exert their neuroprotective effects by acting as endogenous ligands for G-protein-coupled receptors (GPCRs) and modulating gene expression by inhibiting histone deacetylases (HDACs). **b** Trimethylamine N-oxide (TMAO) promotes microglial activation, neuroinflammation, Aβ and tau pathology. **c** Neuroprotective bile acids (BAs), including UDCA and TUDCA, inhibit neuroinflammation via direct and indirect pathways. In the direct pathway, UDCA and TUDCA activate the nuclear receptor Farnesoid X receptor (FXR) and membrane receptor Takeda G-protein-coupled receptor 5 (TGR5) found in microglia and neurons. In the indirect pathway, UDCA and TUDCA provide signals to the central nervous system indirectly via intestinal TGR5-dependent glucagon-like peptide-1 (GLP-1) pathway and intestinal FXR-dependent fibroblast growth factor 15 or 19 (FGF15/19) pathway. **d** Tryptophan and indole derivatives activate microglial aryl hydrocarbon receptor (AHR) signaling to inhibit microglial activation and neuroinflammation. **e** Polyunsaturated fatty acids (PUFAs): omega-3 fatty acids exhibit neuroprotective effects in Alzheimer’s disease, whereas omega-6 fatty acid arachidonic acid and its pro-inflammatory metabolites induce microglial activation. This figure was created with BioRender (https://biorender.com/)

Studies have demonstrated the neuroprotective effects of sodium butyrate in 5xFAD mice by inhibiting microglial activation and promoting synaptic plasticity (Fig. 3a).^299,300^ Moreover, probiotic and prebiotic interventions aimed at elevating SCFA levels have demonstrated neuroprotective effects in AD mouse models by inhibiting glial activation and Aβ deposition.^301–306^ Elevating butyrate through probiotic intervention (*Clostridium butyricum*) has been shown to inhibit microglial activation and reduce the levels of levels pro-inflammatory cytokines in APP/PS1 mice. Furthermore, butyrate exerts anti-inflammatory effects by downregulating the levels of cyclooxygenase-2 (COX-2) and CD11b, and suppressing NF-κB signaling in Aβ-induced BV-2 cells.^303^ Notably, an oral combination therapy (AMX0035) comprising sodium phenylbutyrate and tauroursodeoxycholic acid (TUDCA) is currently undergoing a phase II clinical trial to evaluate its safety and biological activity in AD patients [NCT03533257].

However, conflicting findings regarding the roles of SCFAs in AD have also been reported. A recent population-based study has revealed a positive association between serum propionic acid and cognitive decline in older adults, with potential mediation by hypercholesterolemia and diabetes.^307^ Interestingly, it has been demonstrated that SCFAs induce microglial activation and worsen Aβ pathology in both SPF and GF APP/PS1 mice.^199^ Furthermore, SCFA supplementation has been found to trigger C/EBPβ/AEP signaling activation and induce cognitive impairment in GF 3xTg-AD mice.^196^ The C/EBPβ is an inflammation-regulated transcription factor that regulates the expression of pro-inflammatory genes, thereby contributing to the pathogenesis of AD.^204,308,309^ The AEP is a lysosomal cysteine protease that cleaves tau at N255 and N368 residues, and amyloid precursor protein (APP) at N373 and N585 residues, resulting in amyloidogenic fragmentation and tau hyperphosphorylation.^310,311^ In GF 5xFAD mice, the administration of acetate aggravates hippocampal Aβ deposition by disrupting microglial phagocytosis of Aβ.^190^ The detrimental effects of SCFAs are similarly evident in a tauopathy mouse model. A recent study demonstrated that SCFA supplementation in GF TE4 mice mitigated the neuroprotective effects of GF rearing, resulting in increased gliosis and tau pathology. Conversely, the depletion of SCFAs-producing bacteria using antibiotic treatment conferred protection against tau-mediated neurodegeneration and neuroinflammation in TE3 mice.^56^ A recent study reported that intermittent fasting is effective in alleviating reactive microgliosis and astrogliosis, Aβ deposition, and cognitive impairment of 5xFAD mice by remodeling the microbiota–gut–brain axis. However, metabolomic analysis of cecal contents found that butyric acid was significantly downregulated in response to intermittent fasting, as compared to mice that were fed ad libitum.^312^

The considerable heterogeneity observed across distinct mouse models of AD, each characterized by distinct pathological pathways, poses a significant challenge in anticipating the implications of SCFAs. Moreover, the majority of studies that reported the detrimental effects of SCFAs were conducted using GF mice,^56,190,196,199^ which are functionally and structurally abnormal across various physiological functions.^313^ Thus, caution is warranted when extrapolating these findings to human diseases. In conclusion, it is evident that further investigation is warranted to elucidate the multifaceted nature and contextual significance of SCFAs in AD.

#### Parkinson’s disease

**Fig. 4:**
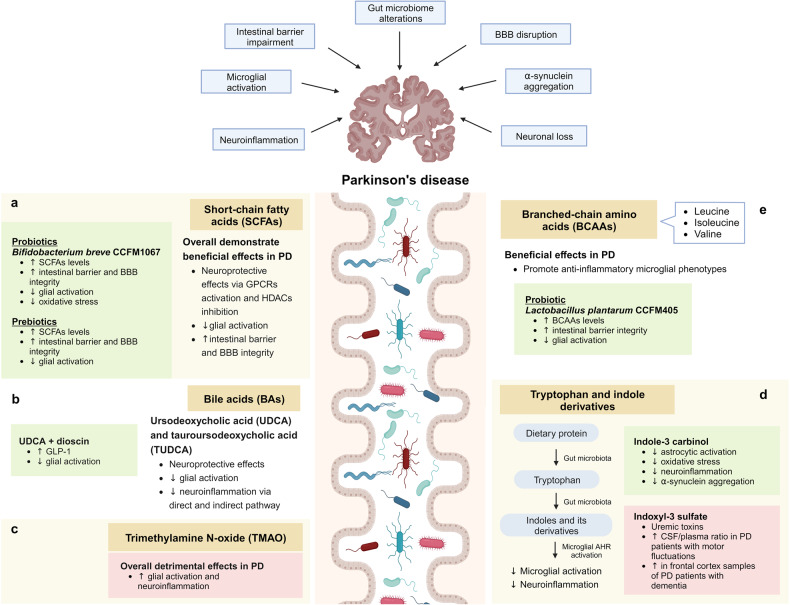
Microbiota–gut–brain axis in Parkinson’s disease. **a** Short-chain fatty acids (SCFAs) exert their neuroprotective effects by acting as endogenous ligands for G-protein-coupled receptors (GPCRs) and modulating gene expression by inhibiting histone deacetylases (HDACs). **b** Neuroprotective bile acids (BAs), including UDCA and TUDCA, inhibit neuroinflammation via direct and indirect pathways. In the direct pathway, UDCA and TUDCA activate the nuclear receptor Farnesoid X receptor (FXR) and membrane receptor Takeda G-protein-coupled receptor 5 (TGR5) found in microglia and neurons. In the indirect pathway, UDCA and TUDCA provide signals to the central nervous system indirectly via intestinal TGR5-dependent glucagon-like peptide-1 (GLP-1) pathway and intestinal FXR-dependent fibroblast growth factor 15 or 19 (FGF15/19) pathway. **c** Trimethylamine N-oxide (TMAO) promotes microglial activation and neuroinflammation. However, contradictory findings have been reported regarding the roles of TMAO in PD. **d** Tryptophan and indole derivatives activate microglial aryl hydrocarbon receptor (AHR) signaling to inhibit microglial activation and neuroinflammation. **e** Branched-chain amino acids (BCAAs) promote anti-inflammatory microglial phenotypes. This figure was created with BioRender (https://biorender.com/)

As with AD described previously, the roles of SCFAs in PD appear to be context-dependent and remain incompletely comprehended. Nevertheless, the majority of studies support the beneficial effects of SCFAs on microglial functions in the context of PD (Fig. 4a). Emerging evidence has revealed that epigenetic perturbation is an important contributor to PD, positioning it as a promising target for potential therapeutic interventions.^286,314^ For example, HDAC5 inhibition is effective in attenuating microglial activation and PD-related pathologies in 6-hydroxydopamine (6-OHDA)-lesioned rats.^315^ On the other hand, the activation of GPCRs has been demonstrated to exhibit neuroprotective properties in PD mouse models. Notably, the administration of probiotic *Clostridium butyricum* triggers the activation of colonic GPR41/43, resulting in the inhibition of microglial activation and mitigation of PD-related pathologies in MPTP-treated mice.^316^ Moreover, the activation of GPR41 in enteric neurons using AR420626 has been shown to mimic the neuroprotective effects of propionate in improving motor functions and preventing dopaminergic neuronal loss in 6-OHDA-induced PD mice.^317^

Boosting the levels of SCFAs with prebiotic intervention upregulates the neuroprotective phenotype of microglia in ASO mice, coupled with reduced motor deficits and α-synuclein aggregation in the SN.^318^ Similarly, the administration of probiotic *Bifidobacterium breve* CCFM1067 restored the levels of SCFAs in MPTP-induced PD mice, resulting in reduced glial activation, oxidative stress, and motor impairments.^260^ Additionally, studies have demonstrated the neuroprotective effects of sodium butyrate in MPTP-induced PD mice by attenuating microglial reactivity via the inhibition of TLR4/MyD88/NF-κB and MAPK signaling pathway.^319–321^ These findings are in concordance with a substantial body of literature that consistently reports diminished SCFAs-producing species and SCFA levels in prodromal stage of PD and PD patients compared to healthy controls, which may be correlated with the clinical severity of PD.^52,322–331^ The observed reduction in SCFAs in PD has inspired a proof-of-concept study to investigate the potential of prebiotic fibers in PD patients [NCT04512599]. Importantly, the trial reported an increased abundance of SCFAs-producing species and increased plasma SCFA levels, along with improved intestinal barrier integrity and reduced intestinal inflammation.^332^

Nonetheless, discrepant findings on the influence of SCFAs on microglial functions in PD have been documented. Of note, SCFA supplementation in GF ASO mice induced microglial activation, α-synuclein accumulation and motor dysfunction.^55^ Conversely, the reduction of SCFAs in MPTP-induced mice yielded beneficial effects, including alleviation of motor dysfunction, microglial and astrocytic activation in the SN.^217,333^ The intriguing duality of SCFAs in PD warrants further exploration. We speculate that the activation of C/EBPβ/AEP signaling might serve as a plausible mechanism underlying the detrimental effects of SCFAs, as demonstrated in GF 3xTg-AD mice.^196^ A recent study found that the C/EBPβ/AEP signaling is age-dependently activated in human α-synuclein transgenic mice and PD patients, which is responsible for mediating microglial activation and PD pathologies.^334^

In addition, growing evidence is shedding light on the distinct effects of different SCFAs on microglial functions. In particular, acetate has been found to restore microglial homeostasis in GF mice, while propionate and butyrate do not have the same restorative effect.^190^ This is further complicated by a recent study which reported that both reduced fecal propionic acid and butyric acid following low-dose maslinic acid treatment, as well as increased fecal acetic acid following high-dose maslinic acid treatment, demonstrated neuroprotective effects against PD pathologies in MPTP-treated mice. However, only high-dose maslinic acid treatment reduced microglial activation and neuroinflammation, and the effects are speculated to be mediated by acetic acid.^335^ On the other hand, a comparison between acetate, propionate, and butyrate in primary microglia has reported an abundant overlap between butyrate and propionate in microglial transcriptomic profile. However, individual SCFA failed to achieve comparable effects as the combined SCFA treatment.^199^ Thus, we recommend future studies to examine the effects of individual SCFA supplementation and combined SCFA supplementation on microglial function in PD mouse models. The beneficial effects of butyrate supplementation on PD pathologies might be attributed to its potent HDAC inhibitory activity and its ability to promote intestinal barrier and BBB integrity.^336–339^

#### Amyotrophic lateral sclerosis

Growing evidence has linked epigenetic dysregulations to ALS.^340^ Notably, genetic knockdown and pharmacological inhibition of HDACs have been shown to ameliorate ALS pathology in different ALS models.^341–344^ Furthermore, studies have identified reduced butyrate-producing species in ALS patients and SOD1^G93A^ mice.^221,222,345,346^

Butyrate supplementation has demonstrated neuroprotective effects in SOD1^G93A^ mice.^222,347^ The administration of butyrate has been shown to increase the abundance of butyrate-producing *Butyrivibrio fibrisolvens*, restore Paneth cell homeostasis and enhance the intestinal barrier integrity of SOD1^G93A^ mice. This is accompanied by reduced SOD1^G93A^ aggregation in the intestine, thereby slowing disease progression and prolonging the lifespan of SOD1^G93A^ mice.^347^ Moreover, butyrate treatment reduces SOD1^G93A^ aggregation and GFAP expression in the colon and lumbar spine of SOD1^G93A^ mice, resulting in improved enteric neuromuscular function.^222^ Using motor neuron-like NSC34 cells with overexpression of hSOD1^G93A^, it was found that butyrate improved mitochondrial bioenergetics by improving mitochondrial network and upregulating the transcription of peroxisome proliferator-activated receptor-gamma coactivator-1α (PGC1α).^348^ The PGC1α signaling is a master regulator of mitochondrial biogenesis and energy metabolism with significant therapeutic relevance in neurodegenerative diseases, including ALS.^349^

### Bile acids regulate neuroinflammation and neurodegeneration

BAs are amphipathic cholesterol metabolites that serve diverse signaling functions. The primary BAs (cholic acid and chenodeoxycholic acid) are synthesized primarily in the liver, and released into the small intestine upon food intake, the majority of which are reabsorbed in the terminal ileum and recycled via enterohepatic circulation. However, non-reabsorbed primary BAs are converted by the gut microbiota into secondary BAs (deoxycholic acid, DCA, and lithocholic acid, LCA) via deconjugation and dehydroxylation, endowing them with new biological activities.^20,350^ This phenomenon is evidenced by the significantly higher concentration of secondary BAs in the intestines and fecal samples of SPF mice compared to GF mice, providing strong evidence for their microbial origin.^351–353^ Moreover, reduced concentrations of DCA and LCA were detected in the intestinal and fecal samples of individuals who had recently used antibiotics, albeit with the caveat of a small sample size.^354^

#### Alzheimer’s disease

Several studies have linked an altered BA profile to AD. Although studies in AD patients have yielded slightly heterogeneous results in BA composition, they have reported a consistent pattern of reduced levels of primary BAs and elevated levels of secondary BAs. Secondary BAs associated with AD and cognitive impairments include DCA, LCA, glycodeoxycholic acid, taurodeoxycholic acid, glycolithocholic acid, and taurolithocholic acid.^355–358^ Moreover, an elevated level of secondary BA taurohyodeoxycholic acid was reported in the serum of GF 3xTg-AD mice receiving FMT from AD patients.^196^ In another study, the administration of a traditional Chinese medicine decoction effectively ameliorated Aβ plaque pathology, neuroinflammation, and cognitive impairment in APP/PS1 mice. These beneficial effects were attributed to the suppression of gut dysbiosis and a reduction in the serum levels of secondary BAs, namely DCA and taurohyodeoxycholic acid.^359^

Nevertheless, it is noteworthy that certain secondary BAs serve significant biological functions, including the regulation of host metabolism, immunity, and resistance against intestinal pathogen expansion.^21,360–364^ More importantly, several secondary BAs have demonstrated pronounced neuroprotective and anti-inflammatory activities, particularly ursodeoxycholic acid (UDCA) and its amidated conjugate, TUDCA (Fig. 3c). They exert anti-inflammatory effects by activating the nuclear receptor Farnesoid X receptor (FXR) and membrane receptor Takeda G-protein-coupled receptor 5 (TGR5), both of which are found in microglia and neurons.^365,366^

The anti-inflammatory activity of TUDCA has been demonstrated in LPS-stimulated primary microglial cells and BV-2 microglial cells.^367–369^ In addition, TUDCA is effective in attenuating microglial reactivity in LPS-treated mice.^367,370,371^ Notably, TUDCA treatment has demonstrated the ability to inhibit Aβ deposition and glial activation in APP/PS1 mice and HFD-fed A7-Tg mice.^372–375^

Moreover, BAs have the potential to modulate the gut–brain axis by maintaining intestinal homeostasis, as TGR5 and FXR signaling are pivotal regulators of intestinal immune response and barrier function.^365,376^ Peripheral BAs provide signals to the CNS indirectly via TGR5-dependent glucagon-like peptide-1 (GLP-1) pathway and the FXR-dependent fibroblast growth factor 15 or 19 (FGF15/19) pathway.^350^ Currently, a phase II clinical trial is underway to examine the safety and biological activity of AMX0035, an oral combination of TUDCA and sodium phenylbutyrate, in the treatment of AD [NCT03533257].

#### Parkinson’s disease

The dysregulation of BA homeostasis has been implicated as a pivotal factor in the pathogenesis of PD. Studies have revealed elevated levels of secondary BAs in PD patients, while findings regarding primary BAs have yielded varying results.^377–380^ An elevated level of primary BA (cholic acid) was identified in the plasma of PD patients.^379,380^ Conversely, a reduction in glycine-conjugated primary BAs (glycocholic acid and glycochenodeoxycholic acid) was reported in post-mortem frontal cortex samples of PD patients, which was associated with the duration of PD diagnosis.^377^ However, another study reported increased levels of glycine- and taurine-conjugated primary BAs in plasma samples of PD patients.^379^ The discrepancy in BA profiles between plasma samples and brain samples warrants further investigation to understand the potential implications for PD pathogenesis. Notably, elevated levels of BAs were detected in the plasma samples of pre-PD patients, indicating that alterations in BA profile manifest many years before the onset of PD.^381^ Moreover, PD patients exhibited reduced plasma levels of neuroprotective BAs, namely UDCA and TUDCA.^379^ This observation aligns with the findings obtained in prodromal PD mice.^382^ Interestingly, a significant elevation in the risk of PD was observed among individuals who underwent cholecystectomy (removal of the gallbladder), a surgical procedure that has been associated with detrimental effects on both gut microbiota and BA composition.^383–385^ It was reported that mice that received FMT from patients with post-cholecystectomy diarrhea exhibited increased levels of secondary BAs, specifically DCA, LCA, and hyodeoxycholic acid.^383^

Similar to AD, the administration of UDCA and TUDCA represents a promising therapeutic approach to counteract microglial activation and neuroinflammation in PD (Fig. 4b). Multiple in vivo studies have demonstrated the neuroprotective effects of UDCA and TUDCA in counteracting mitochondrial dysfunction, oxidative stress, and neuroinflammation within PD mouse models.^386–392^ Moreover, the administration of a low-protein high-carbohydrate diet effectively mitigated PD pathologies in MPTP-treated mice by increasing the serum concentrations of TUDCA.^393^ These encouraging results have prompted two clinical trials, NCT03840005 (phase II) and NCT02967250 (phase I), which investigate the application of UDCA in PD patients. UDCA was found to be safe and well-tolerated in PD patients at a daily dose of 30 mg/kg.^394^

Aside from the direct effects of brain BAs, peripheral BAs provide signals to the CNS indirectly via the TGR5-dependent GLP-1 pathway and FXR-dependent FGF15/19 pathway.^350^ Notably, the modification of gut microbiota, BA metabolism and activation of intestinal TGR5 by dioscin treatment led to enhanced secretion of GLP-1 in both the intestine and brain of MPTP-treated mice. This is coupled with reduced glial activation and amelioration of motor deficits. Importantly, the co-administration of UDCA and dioscin further enhanced the neuroprotective effects of dioscin.^390^ GLP-1 is an incretin hormone that plays a pivotal role in stimulating insulin secretion and lowering blood glucose levels, making it highly relevant in the context of neurodegenerative diseases.^395^ The administration of GLP-1 receptor agonist inhibited microglial activation, prevented microglial-mediated conversion of astrocytes into the neurotoxic A1 phenotype, and effectively protected against α-synucleinopathy-induced neurodegeneration.^396^ Furthermore, the administration of probiotic *Clostridium butyricum* restored the levels of colonic GLP-1 and expression of cerebral GLP-1 receptor in MPTP-treated mice, leading to reduced microglial activation and alleviated motor deficits.^316^

#### Amyotrophic lateral sclerosis

Alterations in BA profiles have been documented in ALS patients and SOD1^G93A^ mice, emphasizing the potential relevance of BAs to ALS pathology.^397,398^ A significant reduction in primary BAs and neuroprotective BA (TUDCA) was recently reported in the fecal samples of ALS patients with cognitive impairment compared to those with normal cognition.^398^ The existing evidence strongly supports the neuroprotective effects of TUDCA in ALS. TUDCA has been found to confer protection against cyclopiazonic acid-induced degeneration in both mouse and human stem cell-derived hSOD1^G93A^ motor neurons. Furthermore, TUDCA enhances neuromuscular junction innervation in the *tibialis anterior muscle* of early-stage hSOD1^G93A^ mice.^399^

Notably, an oral combination of TUDCA and sodium phenylbutyrate (AMX0035) is effective in slowing functional decline and prolonging the overall survival of ALS patients.^400–402^ The promising data from the phase II CENTAUR trial has recently led to its approval by the US FDA for the treatment of ALS.^219^ The phase III PHOENIX trial of this combination is ongoing [NCT05021536]. In addition, a phase III clinical trial is underway to evaluate the safety and efficacy of TUDCA as an add-on treatment to riluzole in ALS patients [NCT03800524].

### Trimethylamine N-oxide promotes neuroinflammation and neurodegeneration

Trimethylamine N-oxide (TMAO) is a metabolite derived from dietary choline, betaine, and l-carnitine via a two-step pathway. Dietary choline is initially metabolized by the gut microbiota into trimethylamine (TMA), which is then absorbed and further oxidized in the liver into TMAO.^403^ A functional gut microbiota is required for the accumulation of TMAO in the brain tissue of aged mice, as aged GF mice displayed lower TMAO than aged SPF mice.^404^ This is consistent with another metabolomic analysis which reported significantly reduced levels of TMAO in the brain, serum, and feces of GF mice compared to conventionally raised mice, corroborating its microbial origin.^405^

#### Alzheimer’s disease

Two recent metabolomic studies have reported elevated TMAO levels in the brains of aged mice.^406,407^ In addition, the levels of TMAO in the plasma and brain of 18-month-old 3xTg-AD mice are markedly higher than those in 8-month-old mice.^408^ Importantly, elevated levels of TMAO have been observed in the plasma and cerebrospinal fluid (CSF) of individuals with MCI and AD.^409,410^ Several studies have shown that TMAO has the ability to traverse the BBB and contribute to neurodegeneration by inducing microglial and astrocytic activation.^411–414^

TMAO supplementation has been shown to induce cognitive impairment in APP/PS1 mice by promoting neuroinflammation, Aβ and tau pathology (Fig. 3b). Interestingly, the study also found that TMAO supplementation disrupted the integrity of the intestinal barrier and BBB.^414^ Moreover, TMAO induces inflammation and aggravates Aβ and tau pathology in D-galactose/AlCl_3_-induced AD mice by activating the PI3K/AKT/mTOR signaling pathway.^415^ Conversely, the reduction of TMAO following the administration of 3,3-dimethyl-1-butanol alleviated cognitive impairment of APP/PS1 mice by attenuating Aβ pathology and neuroinflammation.^416^ The administration of probiotic *Lactobacillus plantarum* is effective in remodeling the gut microbiota and reducing TMAO levels of APP/PS1 mice, resulting in amelioration of neuroinflammation and neurodegeneration.^417^ In addition, physical exercise has been shown to reduce the serum concentrations of TMAO, TMA, and betaine in APP/PS1 mice, conferring protection against neuroinflammation and AD pathologies.^414^

#### Parkinson’s disease

An evident increase in TMAO synthesis was detected in the post-mortem frontal cortex of PD patients with dementia, in comparison to patients with normal cognitive function and MCI.^377^ Moreover, elevated plasma TMAO levels in PD patients are correlated with disease severity and motor symptom progression. Patients with higher baseline TMAO levels are at higher risk of experiencing deterioration in motor symptoms and cognitive function.^418^ However, contradictory findings have been reported regarding the roles of TMAO in PD. A study on drug-naïve early-stage PD patients found that the plasma TMAO levels were lower in PD patients compared to healthy controls. Moreover, patients with lower plasma TMAO levels are associated with a higher rate of increase in levodopa-equivalent dose, as well as a higher risk of dementia conversion.^419^ Further research is needed to clarify the contribution of TMAO in the pathogenesis of PD.

Nevertheless, the existing evidence from PD models has indicated potential detrimental effects of TMAO (Fig. 4c). TMAO pre-treatment has been shown to worsen MPTP-induced microglial and astrocytic activation in the striatum, SN, and hippocampus of MPTP-treated mice.^412,413^ Using midbrain organoid models, it was found that TMAO induced pathological changes similar to PD, including loss of dopaminergic neurons, astrocytic activation, and phosphorylation of α-synuclein.^420^

### Tryptophan and indole derivatives regulate neuroinflammation and neurodegeneration

Amino acids are vital precursors to numerous bioactive molecules, including neurotransmitters and neuromodulators, making them indispensable for optimal brain function and health.^421^ Growing evidence has elucidated the pivotal roles of gut microbiota in the metabolism and utilization of essential amino acids, particularly tryptophan. Alterations in tryptophan metabolism have been implicated in the pathogenesis of neurodegenerative diseases, including AD, PD, ALS, and HD. Tryptophan is an essential amino acid obtained through dietary sources and acts as a biosynthetic precursor to several microbial and host metabolites, including indole and its derivatives.^422–424^ Notably, significantly higher concentrations of indole derivatives were detected in the gut, serum, and brain of SPF mice compared to GF mice, thereby corroborating their microbial origin.^351,405^

The indole derivatives exert a wide range of immunological activities by binding to the AHR. The AHR is a ligand-dependent transcription factor expressed by epithelial cells, immune cells, microglia, and astrocytes.^247,248,425^ Previous studies on multiple sclerosis mouse models have demonstrated that the AHR acts as a negative regulator of NF-κB activation, and the specific deletions of *Ahr* in astrocytes and microglia resulted in CNS inflammation and neurodegeneration.^247,248^

#### Alzheimer’s disease

An altered composition of indole-producing bacteria was observed in APP/PS1 mice, concomitant with compromised intestinal barrier integrity and cognitive impairment. The administration of an indole mixture (indole, indole-3-acetic acid, and indole-3-propionic acid) attenuated the microglial reactivity and neuroinflammation by activating microglial AHR signaling and inhibiting NLRP3 inflammasomes. Consequently, the treatment led to reduced Aβ deposition, diminished tau hyperphosphorylation and improved cognitive function (Fig. 3d).^426^ Moreover, the administration of high-tryptophan diet proved effective in alleviating gut dysbiosis and inhibiting microglial reactivity in APP/PS1 mice by activating central AHR signaling and inhibiting NF-κB signaling.^427^

On the other hand, a significantly higher concentration of indoxyl-3-sulfate was reported in the brain and serum of GF 3xTg-AD mice receiving FMT from AD patients, when compared to their counterparts receiving healthy control microbiota.^196^ Indoxyl-3-sulfate is a uremic toxin adversely related to several mental and neurological conditions, including anxiety, anorexia nervosa, AD, and PD.^377,428–431^ An in vitro study revealed that it induces oxidative stress and inflammation in primary astrocytes and mixed glial cell cultures.^432^ Further studies are needed to comprehensively understand the roles of different indole derivatives in the pathogenesis of AD.

#### Parkinson’s disease

A recent metagenomic analysis of the gut microbiome of PD patients has revealed a reduction in the tryptophan biosynthesis pathway.^331^ Dietary supplementation of tryptophan has been shown to alleviate inflammation and motor deficits of rotenone‐induced PD rats by inhibiting NF‐κB activation, but the neuroprotective effects were blocked by an AHR inhibitor.^433^ In addition, studies have demonstrated the neuroprotective effects of indole-3 carbinol in rotenone-induced PD rats and LPS-treated rats (Fig. 4d).^434,435^ Notably, indole-3-carbinol effectively prevents rotenone-induced α-synuclein accumulation, astrocytic activation, neuroinflammation, and motor dysfunction by activating the SIRT1/AMP-activated protein kinase (AMPK) signaling pathway.^434^ Moreover, indole-3-carbinol is effective in counteracting LPS-induced oxidative stress and neuroinflammation.^435^

On the other hand, the uremic toxin indoxyl sulfate is increased in PD patients.^377,429^ An elevated CSF/plasma ratio of indoxyl sulfate was detected in PD patients, with notably higher concentrations observed in patients experiencing motor fluctuations compared to those without motor fluctuations.^429^ Furthermore, a marked upregulation of indoxyl sulfate was identified in the post-mortem frontal cortex samples of PD patients with dementia, compared to patients with normal cognitive function and MCI.^377^

### Polyunsaturated fatty acids regulate neuroinflammation

Polyunsaturated fatty acids (PUFAs), encompassing omega-3 and omega-6 fatty acids, are essential fatty acids with diverse roles in human physiology, including CNS functioning. These include the regulation of neurogenesis, synaptic plasticity, microglial functions and neuroinflammation. The two predominant PUFAs in the brain are omega-3 fatty acid docosahexaenoic acid (DHA) and omega-6 fatty acid arachidonic acid (AA).^436^ The gut microbiota plays a vital role in the conversion of a subset of PUFAs into bioactive metabolites.^196,437^

#### Alzheimer’s disease

A recent lipidomic analysis of the brains of AD patients has revealed a significant reduction in the ratio of omega-3 to omega-6 fatty acids compared to age-matched cognitively normal individuals. In addition, the study found that omega-3 fatty acids are positively correlated with cognitive function and negatively correlated with Aβ, neurofibrillary tangles burden, and Braak stage. Conversely, the pro-inflammatory metabolites of omega-6 fatty acids are positively correlated with AD pathologies (Fig. 3e).^438^ Indeed, a higher dietary intake of omega-3 fatty acids is associated with a reduced risk of dementia and cognitive decline.^439^ The beneficial effects of omega-3 fatty acids may be attributed to their ability to stimulate microglial phagocytosis of Aβ_42_ and promote the transition of microglia to an anti-inflammatory phenotype, as demonstrated in human CHME-3 microglial cells.^440^

A recent study reported that GF 3xTg-AD mice exhibited reduced genes involved in AA metabolism, along with decreased levels of AA-associated inflammatory enzymes, in comparison to their SPF counterparts. However, FMT from AD patients to GF 3xTg-AD mice remarkably increased the relative abundance of Bacteroides strains involved in PUFA metabolism, resulting in increased concentrations of AA and its metabolites (leukotriene B4, prostaglandin E2, and 12-hydroxyheptadecatrienoic acid). Consequently, the elevated levels of AA-associated metabolites induced microglial activation and the development of AD pathologies in AD-humanized ex-GF 3xTg-AD mice by activating the C/EBPβ/AEP signaling pathway.^196^ Similarly in another study, FMT from AD patients to Thy1-C/EBPβ transgenic mice led to increased levels of prostaglandin E2 and 12-hydroxyheptadecatrienoic acid, resulting in C/EBPβ/AEP pathway activation, microglial activation, and AD pathologies.^441^ Indeed, elevated levels of leukotriene B4 and prostaglandin E2 were recently detected in the post-mortem brains of AD patients.^438^ Interestingly, the co-administration of glyceryl-conjugated prostaglandin E2 and SCFAs additively induced microglial activation in GF 3xTg-AD mice.^196^

#### Amyotrophic lateral sclerosis

PUFAs and their bioactive derivatives are vital regulators of neuronal function and neuroinflammation,^436^ making them highly relevant in the context of ALS. This is evident in two recent metabolomic analyses which revealed significant dysregulation of lipid metabolism, including PUFAs, among ALS patients compared to healthy controls.^442,443^ Notably, a higher plasma level of omega-3 fatty acid α-linolenic acid (ALA) was associated with slower functional decline and longer survival. In addition, higher plasma levels of omega-3 fatty acid eicosapentaenoic acid (EPA) and omega-6 fatty acid linoleic acid (LA) were associated with a lower risk of death, although they did not impact the rate of functional decline.^444^ The protective effects of ALA on ALS are similarly supported by another study, wherein males with higher pre-diagnostic plasma ALA levels are at a lower subsequent risk of developing ALS.^445^ These results are supported by a prospective study that observed a markedly reduced risk of ALS in individuals with higher dietary intakes of omega-3 fatty acids, including ALA and marine omega-3 fatty acids.^446^ On the other hand, an increased risk of ALS is observed in males with higher pre-diagnostic plasma levels of omega-3 fatty acid DHA, and in females with higher pre-diagnostic plasma levels of omega-6 fatty acid AA.^445^

Perturbation in lipid-related metabolism pathways, including AA metabolism, has been identified as a common metabolic signature in a multi-omics analysis of spinal motor neurons (sMNs) derived from four ALS hiPSC lines of various genetic background (*SOD1*^*A4V*^, *C9ORF72*, *TDP-43*^*Q343R*^, and sporadic).^447^ Importantly, the reduction of AA levels using 5-lipoxygenase inhibitor (caffeic acid) is effective in rescuing the reduced percentage of HB9::GFP^+^ cells (a marker of motor neurons) and increased percentage of 7AAD^+^ cells (a marker of cell death) in *C9ORF72* and *SOD1*^*A4V*^ ALS lines. Moreover, caffeic acid treatment is effective in delaying disease onset and prolonging the lifespan of SOD1^G93A^ mice. These beneficial outcomes are attributed to the attenuation of microglial and astrocytic activation, and an increase in the number of motor neurons in the spinal cord.^447^

### Branched-chain amino acids exert anti-inflammatory effects

Branched-chain amino acids (BCAAs) (leucine, isoleucine, and valine) are essential amino acids obtained through diet or gut microbial biosynthesis, as they are not synthesized by humans.^448^ Besides acting as building blocks for protein synthesis, BCAAs play a prominent role in diverse aspects of health and diseases by regulating numerous physiological processes.^449,450^ An early in vitro study has demonstrated the ability of BCAAs to alter microglial phenotypes, particularly towards the anti-inflammatory M2 phenotype (Fig. 4e).^451^

#### Parkinson’s disease

Reduced plasma levels of BCAAs and aromatic amino acids were detected in PD patients, which were significantly correlated with disease severity.^452,453^ This may be related to the reduction in *Prevotella copri* in PD patients,^331,454^ which is the species responsible for BCAA biosynthesis.^448^ A recent study observed reduced serum BCAA levels in rotenone-induced PD mice, accompanied by intestinal dysfunctions, motor deficits, α-synuclein accumulation, and loss of dopaminergic neurons in SN. Conversely, the administration of a high BCAA diet reversed the pathological changes induced by rotenone, along with reduced levels of pro-inflammatory cytokines in the colon and SN.^455^ Furthermore, administration of probiotic *L. plantarum* CCFM405 restored the biosynthesis of BCAAs, restored intestinal barrier integrity, and inhibited microglial and astrocytic activation in rotenone-induced PD mice.^456^

However, we recommend caution against excessive elevation of BCAAs as BCAAs imbalance is associated with detrimental health consequences, including hyperphagia, metabolic disorders, cardiovascular diseases, and reduced lifespan.^450,457^ Another factor warranting consideration is the potential competition between amino acids and levodopa, a commonly prescribed antiparkinsonian drug, in utilizing the intestinal and BBB transporters and consequent interference with levodopa pharmacokinetics.^458,459^

### Nicotinamide mitigates neurodegeneration

Nicotinamide (NAM), also known as niacinamide, is a vitamin B3 derivative and constitutes one of the primary precursors of nicotinamide adenine dinucleotide (NAD^+^).^460,461^ A recent study has revealed a synergistic relationship in which the host-derived NAM is used by the gut microbiome for the synthesis of both NAD^+^ and nicotinic acid. The nicotinic acid is then taken up by the host tissue for NAD^+^ biosynthesis via the Preiss–Handler pathway. Importantly, NAM was not converted to NAD^+^ and nicotinic acid in GF mice and antibiotic-treated mice.^462^

NAD^+^ has been implicated in various aspects of health and disease due to its intricate involvement in numerous cellular processes and metabolic pathways. It is a central regulator of energy metabolism by acting as a coenzyme for redox reactions. Moreover, it serves as a cofactor for a wide variety of enzymes, including those involved in immune response and inflammation. These enzymes include sirtuins, poly(ADP-ribose) polymerase (PARP) protein family, and cyclic ADP-ribose (cADPR) synthases.^460,461^ However, the levels of NAD^+^ undergo progressive decline during aging, leading to the development and progression of age-related diseases.^460^

#### Amyotrophic lateral sclerosis

Neurodegenerative diseases, including ALS, are associated with disrupted NAD^+^ homeostasis and NAD^+^ depletion.^461^ A marked decrease in the levels of NAM has been observed in both the serum and CSF of ALS patients compared to healthy controls. The serum levels of NAM are positively associated with the ALS functional status score.^223^

A notable difference in the metagenomic NAM biosynthetic pathway has been reported between SOD1^G93A^ mice and WT mice. The altered NAM biosynthesis was linked to *A. muciniphila*, which showed a gradual reduction in disease progression in SOD1^G93A^ mice, but not in WT mice. Notably, the administration of *A. muciniphila* significantly improved the motor function and prolonged the lifespan of SOD1^G93A^ mice. These beneficial effects of *A. muciniphila* were attributed to the enhanced NAM biosynthesis, which resulted in increased levels of NAM in the serum and CSF of SOD1^G93A^ mice.^223^ In addition, NAM treatment has been shown to improve the survival of sMNs derived from both sporadic and familial ALS hiPSCs, as well as isogenic iPSC lines harboring SOD1^L144F^ and TDP-43^G298S^ mutations, by rescuing the mitochondrial respiration defects.^463^

## Gut microbiota-related neurotransmitters in neurodegenerative diseases

The complex pathophysiology of neurodegenerative diseases involves multiple neurotransmitter systems dysfunction, including dopaminergic, cholinergic, serotonergic, glutamatergic, and GABAergic systems.^464^ Importantly, the gut microbiome has been shown to regulate brain functions by modulating these neurotransmitter systems.^465^ In this section, we provide an extensive review of the evidence regarding gut microbiota manipulation as a potential therapeutic approach to restore neurotransmitter systems in neurodegenerative diseases.

### Serotonin

Serotonin, also known as 5-hydroxytryptamine (5-HT), is a neurotransmitter that serves a wide range of roles in the brain and gut, more notably its influence on the microbiota the microbiota–gut–brain axis.^466^ Approximately 90% of serotonin is produced and secreted by the gut enterochromaffin cells, which are strongly influenced by the gut microbiota.^33,467,468^ Moreover, the hippocampal serotonin level has been found to be affected by the gut microbiota, possibly by altering the peripheral availability of tryptophan.^469^ Although gut-derived serotonin is unable to cross the BBB, the serotonin precursor (5-hydroxytryptophan) and serotonin derivatives (N-acetylserotonin and melatonin) can cross the BBB to influence the CNS.^34,403^ Interestingly, the artificial elevation of intestinal serotonin levels enriches spore-forming bacteria, particularly *Turicibacter sanguinis*, which is involved in serotonin biosynthesis. These findings indicate a bidirectional host–microbial signaling that regulates gut microbiota colonization via the serotonergic system.^470^

#### Alzheimer’s disease

The dysregulation of the serotonergic system represents a complex pathological process in AD due to its involvement in multiple AD pathologies, including APP processing and Aβ deposition.^471–473^ A recent study reported lower brain serotonin transporter availability and higher cortical Aβ deposition in individuals with MCI compared to healthy controls.^474^ Furthermore, significantly reduced urine and serum serotonin concentrations have been reported in AD patients compared to controls.^475^ Interestingly, the administration of selective serotonin reuptake inhibitors (SSRIs) is effective in suppressing Aβ levels in human and AD mouse models.^471,476,477^

Differences in genes related to the serotonergic system have been reported between GF and SPF 3xTg-AD mice, which suggests higher hippocampal serotonin signaling in the GF mice.^196^ This study corroborates the involvement of gut microbiome in regulating serotonergic system in AD. However, there are currently sparse studies investigating the potential of gut microbiota manipulation to restore the serotonergic system in AD, and the available studies did not consistently show improvements in the serotonergic system. The co-administration of prebiotics fructo-oligosaccharides (FOS) and galacto-oligosaccharides (GOS) increased the concentration of serotonin in the brains of APP/PS1 mice.^478^ However, another study did not observe any improvement in the serotonergic system following prebiotic *Sparassis crispa*-1 polysaccharide administration in d-galactose/AlCl3-induced AD mice.^479^ Nevertheless, the available evidence suggests that probiotics, prebiotics and FMT hold immense potential in AD, as they have shown promising results in modulating the serotonergic system in different neurological disorders.^34,480–483^

#### Parkinson’s disease

Beyond the established dopaminergic dysfunction characterizing PD, growing evidence implicates serotonergic dysfunction in the progression of PD, which is associated with disease burden and may precede motor manifestations or dopaminergic dysfunction.^484,485^ Furthermore, the development of neuropsychiatric symptoms in PD patients, including apathy, anxiety, and depression, is associated with serotonergic dysfunction.^486,487^ In addition, studies have demonstrated the modulation of the serotonergic system by α-synucleinopathy. For instance, the overexpression of human α-synuclein in serotonin receptors in raphe nuclei in mice has been found to impair forebrain serotonin neurotransmission and trigger a depressive-like phenotype.^488^ Conversely, the serotonergic neurotransmission deficits were alleviated following antisense oligonucleotide-induced α-synuclein knockdown.^488,489^

A recent metagenomic analysis of the gut microbiome of PD patients has revealed a dysregulation in the synthesis and metabolism of multiple neurotransmitters, including serotonin. This dysregulation may be attributed to the observed reduction in the tryptophan biosynthesis pathway and sporulation genes.^331^ Additionally, a recent study found that healthy mice, upon receiving FMT from PD mice, exhibited impaired motor function and reduced striatal dopamine and serotonin levels.^217^ These findings underscore the potential significance of gut microbiota manipulation to restore the serotonergic system in PD. Notably, prebiotic polymannuronic acid administration led to the elevation of serotonin and its metabolite, 5-hydroxyindoleacetic acid (5-HIAA), in the striatum of MPTP-induced mice.^490^ Moreover, FMT from control mice mitigated MPTP-induced gut dysbiosis and the decline of striatal serotonin and 5-HIAA in recipient mice.^217^ Conversely, recent studies investigating the impact of probiotics on the serotonergic system in PD have shown less favorable outcomes. In a recent randomized controlled trial (RCT), no significant changes in serum serotonin levels were observed following a 3-month adjunctive probiotic *Bifidobacterium animalis* subsp. *lactis* treatment in PD patients receiving conventional regimen (Benserazide and dopamine agonists).^491^ Furthermore, the administration of *L. plantarum* PS128 was ineffective in restoring MPTP-induced serotonin reduction.^492^ Future studies may explore the potential benefits of multi-strain probiotic formulations for achieving the desired serotonergic effects in PD. These studies should emphasize on the importance of informed strain selection, including the potential synergy or additive effects of individual probiotic strains.^493^

### Gamma-aminobutyric acid (GABA)

GABA is the main inhibitory neurotransmitter primarily generated and regulated by astrocytes and neurons. Tonic GABA current is a vital regulator of brain states and cognitive functions, including learning and memory, sensory processing, circadian rhythm, and vigilance state. Moreover, tonic GABA current is integral for normal motor functions, and its dysregulation has been associated with motor symptoms in PD and HD.^494^ The gut microbiome is involved in the production of GABA, as gut microbiota manipulation has been found to impact GABA levels.^495–497^ Several gut microbes have been identified as GABA producers, including the members of the *Bacteroides*, *Bifidobacterium* and *Lactobacillus* genera.^37,495,498–500^

#### Alzheimer’s disease

The AD pathologies, including Aβ and tau pathology, have been shown to induce GABAergic dysfunction and contribute to excitatory and inhibitory (E/I) imbalance.^501–503^ An abnormal increase in tonic inhibition has been reported in APP/PS1 mice and 5xFAD mice due to aberrant release of GABA by reactive astrocytes, leading to impaired synaptic plasticity and memory.^504,505^ However, recent evidence suggests that GABAergic hypoactivation potentiates neurodegeneration and cognitive impairment in AD patients.^502,506^ Further research is warranted to elucidate the observed discrepancies in the role of GABAergic system within AD pathogenesis, including the involvement of astrocytes.

A recent study has reported variations in the expression levels of genes related to GABAergic system between GF and SPF 3xTg-AD mice, suggesting an increase in hippocampal GABA production and GABA receptors in the GF counterparts.^196^ These findings suggest a potential avenue for manipulating the gut microbiota to address GABAergic dysfunction in AD. In an initial investigation with limited sample size, it was reported that FMT from an AD patient resulted in a reduction of GABA levels in the fecal metabolites of recipient mice.^507^ Conversely, yFMT effectively restored hippocampal GABA levels in aged recipient mice towards the levels observed in young mice, coinciding with improvements in cognitive behavior.^210^ Moreover, the administration of prebiotic *Sparassis crispa*-1 polysaccharide increased the hippocampal levels of GABA in D-galactose/AlCl_3_-induced AD mice, resulting in improved cognitive function.^479^ Future studies employing larger sample sizes and AD mouse models could provide valuable insights into the potential therapeutic implications of modulating the gut microbiome to target the GABAergic system in AD.

#### Parkinson’s disease

Growing evidence has reported alterations in the GABAergic system in PD, which are associated with motor symptoms, psychomotor symptoms, axial symptoms, cognitive impairment, olfactory dysfunction, and visual hallucinations.^508–510^ An alteration in the synthesis and metabolism of GABA was recently reported in a metagenomic analysis of the PD gut microbiome.^331^ Recent studies have explored the potential therapeutic role of probiotics and prebiotics in addressing these underlying pathologies. The administration of probiotic *Pediococcus pentosaceus* and prebiotic polymannuronic acid has shown promise in alleviating gut dysbiosis and reducing GABA levels in the brains of MPTP-induced mice, resulting in the amelioration of motor dysfunction and neuronal degeneration.^490,511^ A recent RCT found that the co-administration of probiotic and conventional regimen in PD patients increased the abundance of species-level genome bins (SGBs) involved in GABA synthesis and reduced the abundance of SGBs encoding GABA degradation, as compared to PD patients receiving conventional regimen alone.^491^

### Dopamine

Dopamine is a multifaceted neurotransmitter involved in the regulation of learning, motivation, motor, and cognitive control, in concert with other neurotransmitters.^512–516^ Disruption of dopaminergic transmission leads to many debilitating CNS disorders, including PD.^517^ Accumulating evidence underscores the regulatory role of the gut microbiome in dopamine signaling.^518^ A recent study found that the exercise-triggered striatal dopamine response was blunted in antibiotic-treated mice due to an increase in the levels of monoamine oxidase, the enzyme responsible for dopamine degradation. Conversely, GF mice receiving FMT from vigorous mice exhibited elevations in dopamine levels.^48^

#### Parkinson’s disease

Aside from the aggregation of misfolded α-synuclein in the intracellular Lewy bodies, a major neuropathological hallmark of PD is the loss of dopaminergic neurons within the SN.^519^ Dopaminergic deficiency underlies the cardinal motor features of PD, including bradykinesia, rigidity, resting tremor, and postural instability.^520^ Thus, the mainstay pharmacological treatments for the management of PD motor symptoms are predominantly dopamine-related interventions, including levodopa preparations, dopamine agonists, and monoamine oxidase-B inhibitors.^519^

The gut microbiome influences multiple aspects of dopaminergic system within PD pathophysiology, as well as the efficacy of dopamine-related drugs.^518,521–523^ This influence is exemplified by a study in which FMT from PD mice reduced striatal levels of dopamine and its metabolites in recipient mice, accompanied by impaired motor function.^217^ Moreover, metagenomic analysis of the gut microbiome in PD patients has detected a reduced capacity to generate dopamine precursors, particularly tyrosine.^331^ Several gut microbes have shown detrimental effects on the dopaminergic system in PD mouse models.^215^ For instance, the administration of *Proteus mirabilis* during the premotor phase of MPTP-treated mice aggravated striatal dopaminergic neuronal damage and motor symptoms.^524^

A substantial body of literature has demonstrated the potential of microbiome-based therapeutics in alleviating dopaminergic damage and motor deficits in PD mouse models.^217,261,321,393,492,525,526^ Adjunctive probiotic administration was found to increase serum dopamine levels and improve motor function in PD patients receiving conventional regimen, compared to patients receiving conventional regimen alone.^491^ Moreover, the administration of berberine has been shown to stimulate the production of dopa and dopamine in the gut microbiota by activating tyrosine hydroxylase and dopa decarboxylase. Dopa, in turn, crosses the BBB and is converted into dopamine. Consequently, the increased levels of dopamine in the brain protected against MPTP-induced motor deficits.^36^

### Acetylcholine

Acetylcholine is a major neurotransmitter released by cholinergic neurons. The cholinergic signaling is responsible for coordinating various cognitive processes in the brain, including memory, learning, attention, sleep, and other higher brain functions.^527^ Increasing evidence is elucidating the complex interactions between the gut microbiome and cholinergic signaling. A recent study found that antibiotic-induced gut dysbiosis and microglial activation resulted in marked reductions in hippocampal synaptic transmission and cholinergic gamma oscillations.^193^ A possible mechanism underlying the antibiotic-induced cholinergic dysfunction is the elevated activity of corticohippocampal acetylcholinesterase (AChE), which leads to increased acetylcholine breakdown.^528,529^ Moreover, the disruption of the gut microbiome in mice fed a high-fat high-sugar diet resulted in increased AChE expression in the brain.^530^

#### Alzheimer’s disease

The significance of cholinergic signaling in AD was long recognized.^531^ A hallmark of cognitive impairment is the loss of basal forebrain cholinergic neurons, which is driven by APP processing, Aβ deposition, tau hyperphosphorylation and dysregulated immune response.^527^ At present, there is a limited number of studies examining the potential of microbiome-based therapeutics for modulating the cholinergic system in AD, but the evidence available supports their beneficial effects on the cholinergic system. Notably, the administration of probiotic *L plantarum* MTCC1325 restored the levels of acetylcholine in the brains of D-galactose-induced AD rats by reducing AChE activity.^532^ Similarly, another study found that prebiotic FOS from *Morinda officinalis* was effective in restoring acetylcholine in the brains of d-galactose-induced AD rats by reducing AChE levels.^533^ Further investigation into the neuroprotective potential of microbiome-based therapeutics in the cholinergic system in other AD animal models is warranted.

### Glutamate

The excitatory neurotransmitter glutamate is a critical regulator of neuronal excitability and synaptic plasticity, which are integral for synaptic transmission, learning, memory, and cognitive function.^534^ Increasing evidence also points towards the role of glutamate, released by astrocytes, as a gliotransmitter to regulate synaptic transmission and plasticity.^535–537^ However, excessive glutamate levels trigger neuronal death through excitotoxicity and have been implicated in the pathogenesis of neurodegenerative diseases.^534^ Similar to the neurotransmitter systems discussed previously, glutamatergic signaling is influenced by the gut microbiome.^465^ The influence of gut microbiome on glutamatergic signaling has been demonstrated across rodent models of different neurological disorders, including schizophrenia,^538^ depression,^44^ AD,^196^ and PD.^331^

#### Parkinson’s disease

Aberrant glutamatergic neurotransmission represents a key contributing factor to neurodegeneration and PD.^377,510,539^ However, our understanding of the influence of the gut microbiome on glutamate in PD remains limited, and the studies available primarily examine glutamate within the gut microbiome. Although PD is generally associated with glutamatergic hyperactivity, a recent metagenomic analysis of PD gut microbiome revealed a reduction in glutamate/glutamine synthesis genes and pathways, along with an increase in glutamate degradation pathway.^331^ These findings are in agreement with the results of an earlier meta-analysis of the PD gut microbiome.^540^ However, it is noteworthy that glutamate does not readily cross the BBB, which may limit its direct influence on brain glutamate levels.^541,542^ On the contrary, several transporters for glutamine, the direct precursor of glutamate, have been identified.^543^ Notably, the combined administration of probiotics with conventional regimen led to a reduction in serum glutamine concentrations in PD patients compared to the placebo group receiving the conventional regimen only.^491^ Further studies are warranted to examine the potential and underlying mechanisms of microbiome-based therapeutics in modulating glutamatergic signaling in PD.

## Gut hormones in neurodegenerative diseases

The hormone-producing enteroendocrine cells (EECs) are specialized epithelial cells found throughout the GI tract.^544,545^ More than 20 gut hormones with overlapping targets and actions have been identified. Once released, the gut hormones exert local effects on neighboring cells within the mucosa and neuronal networks, as well as systemic effects on distant organs, including the CNS.^544,546^ Notably, the gut microbiota-derived metabolites, including SCFAs, secondary BAs, and indoles, have been demonstrated to modulate gut hormone secretion from EECs.^546,547^ In this section, we review gut hormones that have demonstrated significance in neurodegenerative diseases, specifically ghrelin, leptin, and GLP-1.

### Ghrelin

Ghrelin is an octanoylated peptide hormone that exerts its biological activity by acting as an endogenous ligand for the growth hormone secretagogue receptor (GHSR), more commonly known as the ghrelin receptor. The highest expression of ghrelin receptors is found within the brain.^544,548^ The central ghrelin signaling plays a pivotal role in diverse physiological functions, including the regulation of food intake, hippocampal synaptic plasticity and neurogenesis, anxiety, depression, and cognitive function.^544,549–551^ Notably, age-related reductions in ghrelin signaling have been associated with a decline in cognitive function.^551^ The gut microbiome, along with its metabolites, has been reported to modulate ghrelin secretion and CNS functions.^552,553^

#### Alzheimer’s disease

Accumulating evidence has indicated dysfunctional ghrelin signaling in AD. It was found that Aβ binds to and inhibits hippocampal GHSR1α activity in 5xFAD mice, resulting in synaptic deficits and cognitive decline.^554^ In addition, plasma ghrelin levels reduce gradually in 3xTg-AD mice with aging, which is reversed by the administration of multi-strain probiotics formulation (SLAB51), leading to the attenuation of cognitive impairment.^555^ A recent study in the elderly reported an age-dependent reduction in plasma ghrelin and an elevated ratio of plasma liver-expressed antimicrobial peptide 2 (LEAP2)/ghrelin, which is associated with cognitive impairment. Subsequently, the study found that reducing the plasma LEAP2/ghrelin ratio reversed cognitive deficits in aged mice by restoring hippocampal neurogenesis, alleviating synaptic loss, and inhibiting neuroinflammation.^551^ Moreover, the activation of ghrelin signaling has been shown to attenuate microglial activation in mouse models of normal aging, accelerated aging and AD.^556,557^ The neuroprotective effects of ghrelin might be attributed to its ability to impact age-related pathways, including sirtuin-1 activation, with studies demonstrating its ability to prolong lifespan in different mouse models of aging.^556,558^

#### Parkinson’s disease

The ghrelin receptors are expressed in various brain regions, including the SN. Ghrelin participates in the regulation of motor functions by modulating the dopaminergic neuronal excitability within the SN.^559,560^ Additionally, ghrelin promotes the differentiation of midbrain neural stem cells into dopaminergic neurons by activating the Wnt/β-catenin pathway.^561^ Conversely, the inhibition of central GHSR1α activation via intra-SN administration of a selective GHSR1α inhibitor resulted in impaired motor coordination.^562^ Evidence from PD patients has identified reduced plasma ghrelin levels in the fasting state,^563,564^ as well as an impaired ghrelin response and secretion in the postprandial state.^564,565^ These findings have provided a pivotal basis for further investigations into the neuroprotective effects of ghrelin in PD. Indeed, accumulating evidence has demonstrated the neuroprotective effects of ghrelin in multiple rodent models of PD, including MPTP-induced PD mice,^560,566–568^ 6-OHDA-induced PD rats,^569,570^ and A53T α-synuclein transgenic mice.^571^ These beneficial effects are attributed to its ability to promote autophagy, enhance mitochondrial biogenesis and bioenergetics, mitigate neuroinflammation, and inhibit apoptosis.^560,566–569^

### Leptin

Leptin is an adipocyte-derived peptide hormone produced by the white adipose tissue. It conveys metabolic information to the leptin receptors primarily expressed in the CNS to regulate energy homeostasis and immune functions.^572,573^ Notably, impaired brain energetics is a core feature across neurodegenerative diseases, including AD, PD, ALS, FTD, and HD.^574^ Leptin signaling is involved in multiple signaling pathways, including the Janus kinase 2 (JAK2)/signal transducer and activator of transcription 3 (STAT3) pathway, PI3K/AKT pathway, mTOR pathway, and AMPK pathway.^575^ These pathways are highly relevant in the context of aging, neuroinflammation, and neurodegeneration.^245,576^

Recent evidence has demonstrated an association between adiposity and reduced cognitive function,^577–579^ suggesting that leptin signaling represents a target to ameliorate cognitive impairment. Notably, several studies have demonstrated the significant role of gut microbiome in modulating leptin signaling and adiposity.^580–583^ In addition, a recent meta-analysis of 16 RCTs has revealed that the administration of probiotics or synbiotics (prebiotics + probiotics) leads to a significant reduction in circulating leptin levels.^584^

#### Alzheimer’s disease

Leptin is generally considered beneficial for cognitive function, as studies have consistently shown that individuals with higher leptin levels are associated with a reduced risk of dementia and AD.^585–587^ This is supported by a recent meta-analysis that has reported reduced blood leptin levels in AD patients compared to cognitively normal individuals.^587^ Moreover, leptin is involved in counteracting Aβ pathology and tau phosphorylation to prevent synaptic dysfunction.^588,589^ This is evident in a recent study that has revealed a robust association between low plasma leptin and increased CSF Aβ concentration in patients with cognitive impairment, which indicates increased brain amyloid deposition.^590^ Notably, the elevation of plasma leptin levels via chronic administration of multi-strain probiotics has been shown to ameliorate Aβ pathology in 3xTg-AD mice.^555^

#### Parkinson’s disease

Although leptin primarily acts on neurons in the hypothalamus, leptin signaling also has an important role in preserving the dopaminergic system.^591–593^ Leptin receptors are widely expressed in several extra-hypothalamic regions, including dopaminergic neurons in SN.^594,595^ However, our understanding of the role of leptin in PD remains incomplete, with limited evidence from clinical studies. Notably, a recent meta-analysis, including only 198 PD patients and 182 controls, found that the serum leptin levels in PD patients were slightly lower compared to those in the control group, although the difference did not reach statistical significance.^596^ These limited findings emphasize the need for more comprehensive research to understand the mechanisms and functions of leptin in the onset, progression, and management of PD.

### Glucagon-like peptide-1

GLP-1 is an incretin hormone produced by the EECs in response to ingested nutrients to stimulate glucose-dependent insulin secretion and suppress appetite.^544^ GLP-1 has pleiotropic actions due to the extensive expression of GLP-1 receptors (GLP-1R) in multiple organs, including microglia and astrocytes across various brain regions, thus is being explored for its potential in neurodegenerative diseases.^574,597,598^ GLP-1R agonists, approved for the treatment of type 2 diabetes and obesity, have demonstrated promising neuroprotective effects in neurodegenerative diseases owing to their ability to suppress neuroinflammation.^598^ The secretion of GLP-1 is modulated by the gut microbiota,^583,599^ as well as multiple gut microbiota-derived metabolites, including SCFAs, secondary BAs, indoles, and PUFAs.^437,547^ Moreover, probiotics and prebiotics are capable of stimulating GLP-1 secretion and upregulating the expression of GLP-1R in the brain.^316,600,601^

#### Alzheimer’s disease

Diabetes is a well-established risk factor for cognitive impairment and dementia, with dementia now among the leading causes of death in the diabetic population.^602–604^ Given these connections, there is a growing interest in repurposing the anti-diabetic class of drug GLP-1R agonists for AD.^605^ Substantial preclinical evidence supports the neuroprotective effects of GLP-1R agonists on cognition in AD, which is attributed to the ability to reduce Aβ pathology, tau phosphorylation, glial activation, and neuroinflammation.^598,606^ Notably, GLP-1R agonist, NLY01, inhibits Aβ-induced microglial activation via microglial GLP-1R, resulting in reduced reactive astrocyte conversion in both 5xFAD and 3xTg-AD mice.^607^ In addition, the neuroprotective effects of GLP-1R agonists were substantiated by clinical studies that reported a reduction in cognitive impairment and lower dementia incidence among type 2 diabetes patients.^608,609^ These encouraging results have prompted two phase III RCTs, NCT04777396 and NCT04777409, to explore the efficacy of oral GLP-1R agonist semaglutide in early AD. Interestingly, semaglutide treatment has been shown to activate the gut intraepithelial lymphocytes GLP-1R and modulate the gut microbiota, contributing to a reduction in T cell-mediated inflammation.^610^

#### Parkinson’s disease

Diabetes is a well-established risk factor for PD, and has been linked to a faster disease progression, due to the overlapping underlying pathological mechanisms.^611–614^ It has been reported that diabetic patients receiving GLP-1R agonists are associated with a lower risk of developing PD.^615^ The neuroprotective effects of GLP-1R agonists in PD are supported in numerous preclinical studies and have been attributed to their ability to restore insulin signaling, reduce glial activation, neuroinflammation, and oxidative stress.^395^ Similar to the findings in AD mouse models,^607^ the administration of NLY01 prevented microglial activation and reactive astrocyte conversion, effectively protecting against α-synucleinopathy-induced neurodegeneration.^396^ Notably, exenatide, administered at the dose approved for type 2 diabetes treatment, crosses the BBB in appreciable quantities and improves the motor and cognitive function of PD patients.^616^

The amelioration of gut dysbiosis with microbiome-based therapeutics has been shown to restore GLP-1 signaling and attenuate PD pathologies. An engineered GLP-1-producing probiotic strain, *Lactococcus lactis* MG1363-pMG36e-GLP-1, counteracts MPTP-induced neurodegeneration by inhibiting glial activation, oxidative stress, and ferroptosis.^617–619^ In addition, probiotic *C. butyricum* restores colonic GLP-1 levels and upregulates GLP-1R expression in the brains of MPTP-induced mice, accompanied by reduced microglial activation and PD pathologies.^316^ The alleviation of MPTP-induced gut dysbiosis and BAs alterations by dioscin treatment activates the intestinal TGR5 to stimulate GLP-1 secretion and upregulate GLP-1R expression in the brain, thereby attenuating glial activation and neurodegeneration.^390^ Altogether, these studies underscore the potential of gut microbiome interventions to restore GLP-1 signaling and ameliorate PD pathogenesis.

## Points of intervention to improve microbiota–gut–brain axis

In this section, we outline three potential points of interventions to regulate the microbiota–gut–brain axis, namely the intestinal barrier, BBB, and meninges. We provide evidence that dysfunctional barrier integrity induces glial activation and neurodegeneration. Thus, restoring the integrity of these biological barriers holds promise in counteracting dysfunctional glial states in neurodegenerative diseases. Among the biological barriers, targeting the intestinal barrier represents the most promising and straightforward approach, given the direct interactions between gut microbiota and the intestinal barrier. In addition, growing evidence suggests that peripheral immune system may influence neurodegenerative processes directly at the periphery,^620^ Thus, we discuss the evidence of immune regulation by the gut microbiota, specifically focusing on regulatory T (T_reg_) cells and T helper 17 (T_H_17) cells, which are increasingly recognized for their neuroimmune role within the context of gut–brain axis.^621,622^

### Intestinal barrier restoration by microbial metabolites

A pivotal regulator of the microbiota–gut–brain axis is the multi-layered intestinal barrier. The intestinal barrier, comprising the mucus layer, epithelial barrier, and gut vascular barrier, collectively provides the host with excellent protection against external hazards. For a detailed overview of the structure and CNS-related functions of the intestinal barrier, please refer to the comprehensive review by Pellegrini et al.^623^ Gut microbiome alterations and intestinal barrier impairment have been observed in patients with MCI, AD, PD, and ALS patients.^326,540,624–626^ Thus, the restoration of intestinal barrier integrity represents a promising therapeutic strategy to disrupt the cascade of events leading to neurodegeneration.

The mucus layer is a partially penetrable and highly dynamic physical barrier that separates the luminal content from the underlying intestinal layers. Mucins, the primary structural element of the mucus layer, are a family of heavily glycosylated proteins produced and secreted by goblet cells in the epithelium. Apart from its classic GI-related functions, it is increasingly evident that the mucus layer also plays a role in modulating immunity and inflammation by regulating the microbiota composition and the interaction between microbiota and host. The influence of gut microbiota on mucus layer homeostasis has also been revealed.^627,628^ Early evidence has shown that GF mice exhibit characteristics of a defective mucus layer.^629^ As the task of producing large amounts of mucin is challenging to the goblet cells, a controlled unfolded protein response and endoplasmic reticulum (ER) expansion are required to prevent the accumulation of ER stress. An intact gut microbiota contributes to goblet cell maturation and mucus barrier assembly by activating the epithelial cell-specific ER stress sensor ERN2 and its downstream transcription factor X-box binding protein 1 (XBP1).^336^ ERN2 is one of the three major signaling branches of unfolded protein response specifically expressed in mucin-producing goblet cells of gastrointestinal and respiratory tracts. It is responsible for restoring goblet cell proteostasis.^630,631^

**Fig. 5:**
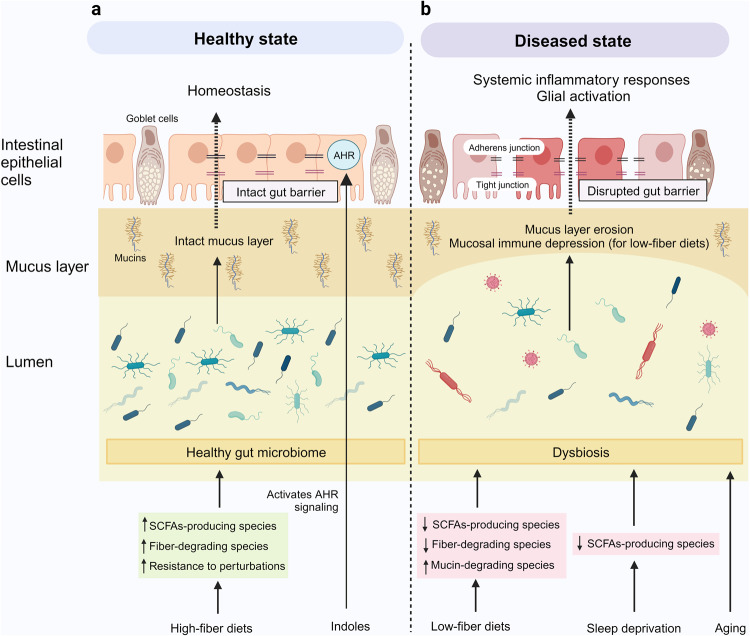
Improving microbiota–gut–brain axis via the intestinal barrier. **a** High-fiber diets contribute to a healthy gut microbiome and enhance intestinal barrier integrity by increasing SCFAs-producing species, and fiber-degrading species and promoting resistance to perturbations. Indole and its derivatives improve intestinal barrier integrity by activating epithelial aryl hydrocarbon receptors (AHR). **b** Low-fiber diets, aging and sleep deprivation contribute to dysbiosis and disrupt intestinal barrier integrity by reducing SCFAs-producing species and fiber-degrading species while increasing mucin-degrading species. Low-fiber diets induce mucosal and systemic immune depression by impairing the metabolic fitness of CD4^+^ T cells. This figure was created with BioRender (https://biorender.com/)

During aging, the intestinal barrier undergoes progressive breakdown, which facilitates the translocation of pro-inflammatory gut microbiota-derived metabolites and pathogenic bacterial products into the bloodstream (Fig. 5). This process triggers elevated systemic inflammatory responses that gradually impair BBB integrity, resulting in CNS inflammation and neurodegeneration.^623^ Microbiome-mediated disruption of the intestinal barrier during aging facilitates excessive translocation of *N*^6^-carboxymethyllysine across the intestinal mucosal barrier into the bloodstream. This increased translocation results in the accumulation of *N*^6^-carboxymethyllysine in the brain, which in turn contributes to microglial dysfunction by inducing oxidative stress and mitochondrial dysfunction.^404^ Notably, FMT from young mice to aged mice restored the intestinal epithelial barrier integrity and attenuated microglial activation in the aged mice.^632^

Interestingly, recent evidence has revealed the adverse effects of neuropathological changes in AD brains on the gut microbiome and intestinal barrier integrity.^633–635^ A recently developed transgenic AD mice model with amyloid and neurofibrillary tangles pathology (ADLP^APT^ mice) manifested altered gut microbiota, chronic intestinal inflammation, increased intestinal barrier permeability, and systemic inflammation. However, daily FMT from WT mice for 4 months successfully transformed the gut microbiota composition of ADLP^APT^ mice, leading to restored circulating Ly6C^hi^ monocytes population, reduced Aβ deposition, tau aggregates, and microglial activation.^635^ Similar findings were reported in another study following intracerebroventricular injection of Aβ_1–42_ oligomers. Aβ_1–42_-treated mice began developing alterations in the composition and diversity of gut microbiota 4 weeks post-surgery. These changes were coupled with elevated intestinal permeability and intestinal inflammation via the inhibition of the cholinergic anti-inflammatory pathway.^634^ These findings underscore the necessity of future studies to delineate the temporal sequence of pathologies.

#### Short-chain fatty acids restore intestinal barrier integrity

The SCFAs produced by the gut microbiota during the fermentation of dietary fiber are critical in the reinforcement of the mucus layer (Fig. 5a). High-fiber intake increases dietary fiber metabolizers and SCFAs producers, which in turn stimulates mucus secretion to maintain a well-structured and intact mucus layer.^636,637^ Importantly, dietary fiber also promotes resistance of human gut microbiota to perturbations by limiting microbial growth and balancing the positive and negative interspecies interactions.^638^ A proof-of-concept trial conducted in PD patients has demonstrated that SCFAs-promoting prebiotic fiber intervention reduced plasma zonulin, indicating improved intestinal barrier integrity.^332^ Moreover, a recent randomized clinical trial reported that a 3-month high-fiber intervention with a Mediterranean diet greatly reduced plasma lipopolysaccharide-binding protein (LBP) and fecal zonulin concentrations in women with an impaired intestinal barrier. The study further performed model-based causal mediation analysis and revealed that the barrier-stabilizing effect of the Mediterranean diet was mediated by the propionate and butyrate.^639^

In contrast, a chronic or intermittent low-fiber diet in gnotobiotic mice stimulates the proliferation and activity of mucin-degrading bacteria, resulting in mucus layer erosion and increased pathogen susceptibility (Fig. 5b).^640^ In addition, feeding mice a western-style diet characterized by low dietary fiber, high fats, and high simple carbohydrates induces depletion of fiber-degrading bacteria, including Bacteroidetes (family S24-7) and Actinobacteria (*Bifidobacterium*). This was accompanied by reduced SCFAs production, reduced inner mucus growth rate, and increased mucus penetrability.^641^ Consumption of a western-style diet also drives the emergence of mutations in *Bacteroides thetaiotaomicron*, an anaerobe specialized in fermenting complex polysaccharides. These mutations promote the degradation of mucin O-glycans in the mouse gut.^642^ Consequently, the increased intestinal permeability facilitates the translocation of bacteria and their byproducts into the mucosa and bloodstream. This leads to aberrant activation of the intestinal and circulating immune and inflammatory cells, resulting in systemic inflammation, neuroinflammation, and neurodegeneration.^623,643^

Restoring the Bacteroidetes of obese mice fed a high-fat and fiber-deficient diet through oat β-glucan supplementation successfully restored the intestinal barrier integrity, including mucus thickness and levels of tight junction (TJ) proteins. In addition, β-glucan supplementation attenuated microglial activation and cognitive impairment in the obese mice. Importantly, the beneficial effects of β-glucan on cognitive function were lost upon antibiotics administration, indicating that the gut microbiota is a vital mediator of cognitive function.^644^ Similarly, reshaping the gut microbiome of HFD-fed mice using dimethyl itaconate restores intestinal barrier integrity by increasing SCFAs-producing bacteria and restoring intestinal immune homeostasis. This is accompanied by alleviation of microglial activation, neuroinflammation, synaptic impairment, and cognitive impairment induced by HFD.^645^

Recently, growing evidence has indicated that sleep deprivation (SD) induces gut dysbiosis and intestinal barrier disruption, leading to microglial activation and cognitive decline (Fig. 5b).^646–648^ Persistent SD (<6 h per night) during midlife is associated with a 30% elevated risk of dementia in a longitudinal study of 7959 participants.^649^ Chronic SD triggers gut microbiota alteration, leading to reduced mucus thickness and reduced levels of TJ proteins in mice colons, via NLRP3 inflammasome activation. This is accompanied by impaired BBB integrity, NLRP3 activation in the brain, microglial activation, and cognitive impairment. Moreover, FMT from chronically sleep-deprived mice to control mice largely recapitulated the pathological changes observed in chronic SD.^646^ Similar detrimental consequences of SD on cognitive function were observed in humans. SD in humans induced depletion of SCFAs-producing species, systemic inflammation via the activation of TLR4/NF-κB signaling pathway. This is coupled with increased serum levels of zonulin and S100β, indicating compromised intestinal barrier and BBB.^647,650^ Interestingly, GF sleep-deprived mice exhibited weaker inflammatory responses, reduced intestinal barrier damage, and milder cognitive impairment as compared to their SPF counterparts, highlighting the crucial role of gut microbiota in mediating SD-induced pathological changes. Indeed, FMT from SD human to GF mice significantly reduced SCFAs levels, promoted both peripheral and central inflammatory responses, and impaired cognitive function.^647^

#### Indole and its derivatives restore intestinal barrier integrity

Other major metabolites involved in the maintenance of intestinal barrier integrity are the indole and its derivatives, which are produced by bacterial fermentation of dietary tryptophan. Tryptophan is an essential amino acid obtained through dietary sources and acts as a biosynthetic precursor to several microbial and host metabolites, including indole.^425,651^ Notably, metagenomic analysis of PD gut microbiome has revealed a reduction in the tryptophan biosynthesis pathway.^331^ Several indole derivatives, including indole-3-ethanol, indole-3-pyruvate, and indole-3-aldehyde, maintain the integrity of the apical junctional complex and improve intestinal barrier integrity by activating the epithelial AHR (Fig. 5a).^652^ The AHR is a ligand-dependent transcription factor widely expressed in the intestinal microenvironment that has a profound influence on the preservation of intestinal barrier integrity.^425^ The AHR signaling participates in the regulation of epithelial cell differentiation and crypt stem cell proliferation. Dietary supplementation with indole-3-carbinol activates the AHR in intestinal epithelial cells to restore intestinal barrier integrity in mice infected with *Citrobacter rodentium*.^653^ Aside from its effects on epithelial AHR, dietary supplementation with indole-3-carbinol also triggers the activation of endothelial AHR in LPS-treated mice. This leads to reduced inflammatory activation in endothelial cells, resulting in improved gut vascular barrier integrity.^654^

A recent study has highlighted significant alterations in indole-producing bacteria in APP/PS1 mice, which were coupled with intestinal barrier dysfunction and cognitive dysfunction. Conversely, oral gavage with a mixture of indole, indole-3-acetic acid, and indole-3-propionic acid reversed the intestinal barrier impairment, reduced Aβ accumulation and tau hyperphosphorylation, leading to improved synaptic plasticity and cognitive function of APP/PS1 mice. In addition, the indoles inhibited microglial activation and NLRP3 inflammasome activation by activating microglial AHR.^426^

### Blood–brain barrier (BBB) restoration by microbial metabolites

The BBB is a complex, multicellular, and dynamic interface crucial for maintaining CNS homeostasis and normal brain function. It stringently regulates the passage of countless molecules, including essential nutrients and deleterious xenobiotic molecules. It is also responsible for the removal of toxic metabolic waste products and endogenous endotoxins from the brain.^655,656^ Importantly, it acts as an immunological barrier by restricting the migration of immune cells into the brain.^657^ The BBB is composed of tightly packed, non-fenestrated endothelial cells linked by TJs and adherens junctions (AJs), which share a common basement membrane with pericytes and astrocytes. These cells, together with neurons and microglia, form the neurovascular unit (NVU) and collectively contribute to the maintenance of BBB integrity.^655,656^

However, the BBB undergoes progressive loss of integrity with age. Compromised BBB initiates the infiltration of peripheral immune cells and triggers microglial and astrocytic activation, ultimately resulting in synaptic and neuronal loss.^658^ The infiltration of blood-derived protein, fibrin, also induces microglial polarization to oxidative stress and neurodegenerative phenotypes in AD and multiple sclerosis mice.^659^ A recent single-nucleus RNA-sequencing analysis of the hippocampus and frontal cortex of AD patients found that 30 of the top 45 AD GWAS hits are expressed in brain vascular cells.^85^ Furthermore, BBB disruption is well characterized in other neurodegenerative diseases, including PD, ALS, FTD, and HD.^658,660^ The BBB disruption in neurodegenerative diseases makes it a clear target of intervention. Despite their anatomical separation, growing evidence has demonstrated the impact of intestinal perturbations on brain health. Ample evidence has shown that gut microbiota-derived metabolites are important regulators of BBB integrity,^661–665^ suggesting that the BBB may be an important communication interface between gut microbiota and glial cells.

#### Short-chain fatty acids improve BBB integrity

**Fig. 6:**
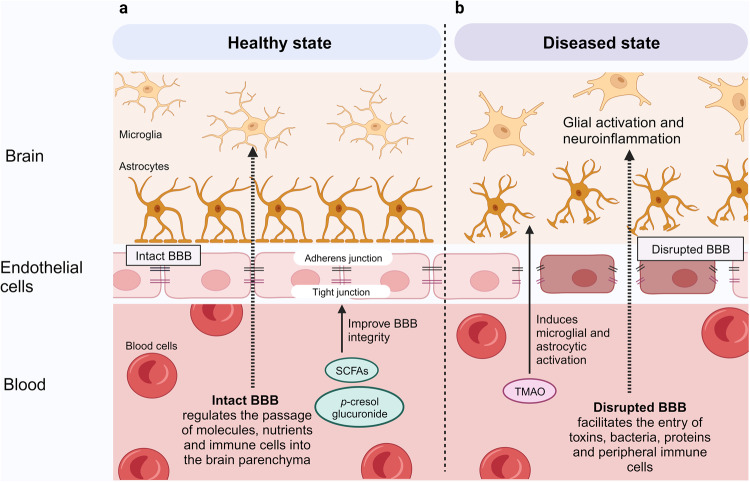
Improving microbiota–gut–brain axis via the blood–brain barrier. **a** SCFAs and *p*-cresol glucuronide improve BBB integrity and prevent glial activation. **b** Elevated levels of trimethylamine-N-oxide (TMAO) has been reported in the plasma and cerebrospinal fluid of individuals with mild cognitive impairment, Alzheimer’s disease (AD) and Parkinson’s disease (PD). TMAO is detrimental to BBB integrity and induces glial activation. This figure was created with BioRender (https://biorender.com/)

SCFAs are the most extensively studied microbial-derived metabolites with considerable influence on BBB integrity (Fig. 6a). An initial investigation revealed that GF mice exhibit increased BBB permeability and compromised TJs as compared to SPF mice. However, the BBB defects in GF mice were restored following mono-colonization with SCFA-producing bacteria or oral gavage with sodium butyrate.^662^ Similar trends were observed following gut microbiota depletion using antibiotic treatment. Antibiotic-treated mice displayed greater BBB permeability and reduced TJ proteins, which were partially ameliorated following FMT from SPF mice.^665^ Studies on in vitro BBB models have elucidated the protective mechanisms of SCFAs.^663,666^ Propionate was found to rescue LPS-induced impairment in the permeability of hCMEC/D3 monolayers by reducing oxidative stress.^663^ Another recent study on bEnd.3 endothelial cells demonstrated that butyrate and propionate improved BBB integrity by regulating the organization of the actin cytoskeleton and increasing the interaction between actin and TJ protein ZO-1. Moreover, butyrate and propionate reversed the LPS-induced reduction in ZO-1 and claudin-5 at the cell–cell junctions and restored the LPS-induced mitochondrial dysfunction.^666^ Further research is warranted to validate the impact of SCFAs on BBB integrity in neurodegenerative disease models.

#### Trimethylamine N-oxide disrupts BBB integrity

Another important metabolite that participates in the regulation of BBB integrity is TMAO (Fig. 6b). Studies have reported elevated levels of TMAO in the plasma and CSF of individuals with MCI, AD, and PD.^409,410,667^ Furthermore, the gene families involved in TMA production were found to be elevated in the PD gut microbiome, leading to increased choline metabolism and subsequent TMA production.^331^ In the brain, TMAO is detrimental to neuronal physiology as it induces neuronal senescence, oxidative stress, and alters synaptic plasticity.^408,668–670^ Moreover, TMAO is also associated with neuroinflammation by inducing microglial and astrocytic activation.^411–414^

However, conflicting results have emerged regarding the role of TMAO on BBB physiology. A recent study found that APP/PS1 mice fed with a TMAO-supplemented diet manifested reduced occludin and ZO-1 expression in the parietal cortex, along with increased microglial and astrocytic activation.^414^ The detrimental effects of TMAO on BBB integrity were also reported in chronic kidney disease patients, using resistance arteries in adipose tissue as a surrogate model of BBB.^671^ However, chronic low-dose TMAO supplementation has been found to prevent LPS-induced BBB alterations and memory impairment in C57Bl/6J mice.^661^ In an in vitro BBB model using hCMEC/D3 cells, it was demonstrated that TMA exposure increased paracellular permeability, whereas TMAO exposure enhanced barrier integrity and trans-endothelial electrical resistance (TEER).

The dichotomous roles of structurally similar TMA and TMAO are intriguing and demand further investigation on the choline-TMA-TMAO pathway. In the context of AD, choline is generally considered beneficial, as a low dietary choline intake has been associated with an elevated risk of dementia and AD.^672^ Lifelong choline supplementation has shown protective effects against Aβ pathology and microglial activation in female APP/PS1 mice.^673^ Conversely, choline deficiency has been found to exacerbate Aβ and tau pathologies in female 3xTg-AD mice.^674^ Furthermore, choline deficiency also impairs glucose metabolism in 3xTg-AD mice and non-transgenic control mice,^674^ which may be relevant to AD as hyperglycemia and diabetes are associated with BBB breakdown and dementia.^675–677^ More studies are needed to understand the mechanisms underlying the choline-TMA-TMAO pathway and determine the optimal gut microbiota manipulation that improves BBB integrity. Notably, a longitudinal study conducted on healthy men has identified ten gut microbial species that are associated with plasma TMAO concentration. Among them, the Bacteroidetes *Alistipes shahii* significantly strengthened the association between red meat/choline intake and plasma TMAO levels.^678^

#### *p*-Cresol glucuronide improves BBB integrity

Another bacterial metabolite involved in the regulation of BBB integrity is *p*-cresol glucuronide (pCG), which is a phenol derived from the microbial metabolism of phenylalanine and tyrosine (Fig. 6a).^664^ Recent evidence has demonstrated that pCG treatment improved the BBB integrity of male C57Bl/6 mice by upregulating several transporter-related pathways and downregulating inflammatory pathways. Subsequent examinations on hCMEC/D3 cells revealed that pCG counteracted the permeabilizing effects of LPS by inhibiting the TLR4 signaling.^664^ Future studies utilizing neurodegenerative disease models are necessary to examine the effects of pCG on BBB integrity during neurodegenerative disease pathology.

### Meninges as an emerging target

The gut–meningeal axis has emerged as another significant aspect of the gut–brain interface that has garnered considerable attention. The meninges are a three-layered membranous barrier, comprising an outer dura mater, middle arachnoid mater, and inner pia mater, that cover the spinal cord and brain parenchyma. Rather than being an inert structural barrier, growing evidence indicates that the meninges actively participate in extensive neuro-immunological crosstalk with the brain. The meninges host diverse innate and adaptive immune cells, which are distributed in varying amounts across the distinct meningeal layers to enable tissue-specific functions.^679^ During aging, the meninges undergo a shift towards pro-inflammatory immune responses, characterized by an increased number of T cells and B cells.^680–683^ Dysfunctional meninges also represent an important contributor to neuroinflammation in neurodegenerative diseases by acting as a checkpoint for T-cell infiltration.^684–686^ Moreover, meningeal lymphatic dysfunction induces microglial activation and acquisition of DAM signature, accompanied by Aβ deposition in the brains of 5xFAD mice.^687^

While the causal relationship between the gut microbiome and meningeal impairment in neurodegenerative diseases has yet to be determined, recent evidence suggests that the microbiota-immune cell dialog has important implications on the meninges. A key mediator of the gut–meningeal axis is the mucosal-associated invariant T (MAIT) cells.^688^ MAIT cells are unconventional, innate-like T cells present abundantly in the human blood, liver, and mucosa. They recognize microbial-derived vitamin B2 (riboflavin) metabolites presented by non-polymorphic major histocompatibility complex (MHC) class I-related (MR1) molecule through their semi-invariant T-cell receptor (TCR).^689,690^ The development and maturation of MAIT cells are dependent on microbiota, as demonstrated by reduced numbers of MAIT cells in GF mice, which can be restored through microbial colonization.^691–693^ In addition, a stressed and unbalanced microbiota in HFD-fed obese mice decreased MAIT cell frequency by reducing MAIT cell agonist ligands. This is accompanied by increased gut inflammation, gut dysbiosis, and compromised gut integrity.^694^

The regulatory role of MAIT cells on meningeal barrier integrity and neuroinflammation was recently demonstrated following the detection of MAIT cells in the meninges and choroid plexus (CP) of mice.^688^ MAIT cells are responsible for preserving meningeal homeostasis by expressing antioxidant molecules, as evidenced by increased oxidative damage and impaired meningeal barrier integrity in MAIT cell-deficient *Mr1*^–/–^ mice compared to their age-matched *Mr1*^+/+^ counterparts. Moreover, *Mr1*^–/–^ mice exhibited extensive microgliosis in the cortex and hippocampus, along with impaired cognitive function. However, these defects were prevented by the adoptive transfer of MAIT cells from WT mice, supporting the protective functions of MAIT cells on the meningeal barrier.^688^ Future studies investigating the effects of gut microbiota manipulations on meningeal MAIT cells and meningeal barrier integrity using neurodegenerative disease mouse models are needed to ascertain the potential of targeting the MAIT cells.

### Restoration of peripheral immune homeostasis

Dysregulation of the immune system, including innate and adaptive immune response, and the resultant chronic inflammation are important drivers of neurodegeneration.^695^ Although peripheral immune cells are generally thought to induce neurodegeneration after CNS infiltration, growing evidence suggests that they may influence neurodegenerative processes directly at the periphery.^620^

The gut microbiome participates in extensive bidirectional communication with the immune system.^622,696^ Notably, certain gut microbiota and their metabolites may stimulate or inhibit the differentiation of naive CD4^+^ T cells into T_H_17 cells. T_H_17 cells are highly abundant at mucosal barriers and play a critical role in regulating tissue homeostasis. A balanced ratio of T_reg_ and T_H_17 cells is vital for maintaining an optimal intestinal immune system.^697^ Unhealthy dietary interventions, such as high-fat, high-sugar, and high-salt interventions, have been shown to negatively impact the gut microbiome and induce intestinal inflammation by impairing T_H_17 cell functions.^698–700^ In addition, the gut microbiome regulates the activity of retinoic acid receptor-related orphan receptor gamma-positive (RORγ^+^) T_reg_ cells, dysregulation of which can lead to diseases such as colitis, food allergy, and colon cancer.^622,701–704^

The SCFAs have been shown to modulate the functions of various immune cells, including T cells, B cells, macrophages, dendritic cells, and innate lymphoid cells.^274,705^ For instance, butyrate has been shown to restrict inflammation by promoting the differentiation of T_reg_ cells and supporting the functions of regulatory B cells.^706–708^ A recent study found that a short-term dietary switch from a fiber-rich diet to a low-fiber feast diet induced a transient depression of mucosal and systemic immunity by impairing the metabolic fitness of both mucosal and peripheral CD4^+^ T cells. These effects are associated with a rapid reduction of SCFAs, impaired mTOR activity, and mitochondrial function of CD4^+^ T cells.^709^ On the other hand, certain secondary BAs exert anti-inflammatory effects by stimulating T_reg_ cell differentiation and inhibiting T_H_17 cell differentiation.^361,363,364,702,710^ While these findings shed light on the relationship between the gut microbiome and immune responses, further studies are needed to elucidate their intricate interplay in neurodegenerative diseases. Emerging evidence has underscored the neuroimmune role of T_reg_ cells and T_H_17 cells in the gut–brain axis, extending to neuropathological conditions such as AD, suggesting potential areas for future research.^621,622^

## Microbiome-based therapeutics

The alterations of the gut microbiome in neurodegenerative disease patients have prompted researchers to explore the clinical applications of microbiome-based therapeutics, including prebiotics, probiotics, and FMT. The rationale is further supported by several studies that demonstrated that the human gut microbiome is a major determinant of plasma metabolome, potentially playing a more dominant role than genetics.^26–28^

### Probiotics and prebiotics

According to The International Scientific Association for Probiotics and Prebiotics (ISAPP), probiotics are defined as “live microorganisms that, when administered in adequate amounts, confer a health benefit on the host”.^711^ The administration of probiotics generally aims to introduce defined microbial strains to stimulate the health-promoting pathways in the microbiome and increase the production of beneficial metabolites.^711,712^ The development of new cultivation and sequencing technologies, along with the expansion of our knowledge of the human gut microbiota landscape has enabled probiotics to move beyond the traditional *Lactobacillus* and *Bifidobacterium* to the next-generation probiotics (NGPs). Examples of NGPs include *A. muciniphila*, *Faecalibacterium prausnitzii*, *Roseburia intestinalis*, and *Bacteroides fragilis*.^712–714^

On the other hand, a prebiotic is defined by ISAPP as “a substrate that is selectively utilized by host microorganisms conferring a health benefit”.^715^ Prebiotics are non-digestible substances that selectively stimulate the growth of beneficial bacteria to increase the production of associated metabolites. The most extensively documented prebiotics include inulin, FOS, and GOS, which promote the growth of *Lactobacillus* or *Bifidobacterium* spp. Nevertheless, technological advances have expanded the targets of prebiotics beyond *Lactobacillus* and *Bifidobacterium*, to other newly identified health-promoting gut microbes, such as *Roseburia, Eubacterium*, and *Faecalibacterium* spp.^715,716^

In this section, we have summarized the findings of in vivo studies investigating the applications of probiotics (Table 1) and prebiotics (Table 2) in neurodegenerative disease models, with a specific focus on their effects on the intestinal barrier, BBB, and glial cells. Overall, these studies have consistently demonstrated the beneficial effects of probiotics and prebiotics on cognitive functions in animal models. This is attributed to their ability to enhance the integrity of the intestinal barrier and BBB, while also promoting the resolution of intestinal inflammation. Probiotics and prebiotics prove effective in re-establishing beneficial gut microbiota and restoring metabolic functions, particularly the SCFA-producing species, resulting in elevated levels of SCFAs. The alleviation of biological barrier impairments, coupled with the reduction in systemic LPS levels, attenuates systemic inflammation and glial activation, resulting in reduced neurodegenerative disease pathology. Furthermore, the administration of probiotics and prebiotics appears to contribute to the restoration of neurotransmitter systems in neurodegenerative disease models.

**Table 1:** In vivo evidence of probiotics in neurodegenerative disease models

Types of probiotics	Subjects	Intervention duration	Key findings	References
Alzheimer’s disease				
* Bifidobacterium longum* NK46	8-month-old 5xFAD mice	6 treatments per week for 8 weeks	• Inhibited LPS-induced NF-κB activation in the colon and hippocampus • Increased TJ protein (claudin-1) expression in the colon • Increased TJ protein (claudin-5) and brain-derived neurotrophic factor (BDNF) expression in the hippocampus • Inhibited Aβ plaque accumulation and microglial activation in the hippocampus • Attenuated cognitive decline	^755^
	19-month-old aged mice	6 treatments per week for 4 weeks		
* Bifidobacterium breve* HNXY26M4	7-month-old APP/PS1 mice	Daily treatment for 12 weeks	• Increased the levels of fecal SCFAs (acetate and butyrate) • Reduced the expression of pro-inflammatory cytokines (TNF-α and IL-1β) in the colon • Increased the expression of TJ proteins (claudin-1 and ZO-1) in the colon • Increased the expression of TJ protein (claudin-5) in the cortex • Reduced Aβ deposition in the brains • Attenuated cognitive impairments	^302^
* Akkermansia muciniphila* GP01	9-month-old APP/PS1 mice	Daily treatment for 6 months	• Restored number of colonic mucus cells and intestinal barrier integrity • Ameliorated lipid metabolism disorders • Reduced Aβ plaque deposition and Aβ levels in the brain • Attenuated cognitive impairments and anxiety-related behaviors	^756^
Mesoporous silica nanoparticle-encapsulated *Bifidobacterium*	7-month-old APP/PS1 mice	Treatment every 4 days for a total of 7 times	• Increased the levels of fecal SCFAs • Reduced colonic mucus damage and increased colonic crypt lengths • Reduced Aβ deposition in the gut, cortex, and hippocampus • Inhibited microglial activation	^301^
* Lactobacillus salivarius*	6-month-old 5xFAD mice	Twice a week treatment for 16 weeks	• Inhibited C/EBPβ/AEP signaling in the gut and brain • Reduced gut leakage • Reduced Aβ pathology in the gut and brain • Reduced APP and Tau proteolytic cleavage by the AEP in the brain • Reduced the levels of IL-6 in the brain • Reduced oxidative stress in the cortex and the hippocampus	^204^
* Lacticaseibacillus paracasei* PS23 (LPPS23)	28-week-old, aged senescence-accelerated mouse prone 8 (SAMP8) mice	Daily treatment for 12 weeks	• Increased intestinal length, reduced intestinal permeability, and increased phagocytic activity • Reduced the levels of pro-inflammatory factors (TNF-α and MCP-1) in intestinal mucosa • Increased IgA levels and reduced IgE levels in intestinal mucosa	^757^
ProBiotic-4 * - Bifidobacterium lactis*, *Lactobacillus casei*, *Bifidobacterium bifidum*, and *Lactobacillus acidophilus*	Aged SAMP8 mice	Daily treatment for 12 weeks	• Attenuated mucus layer atrophy, crypt loss, and villus fracture in the ileum • Increased expression of TJ proteins (claudin-1, occludin, and ZO-1) in the intestine • Reduced plasma levels of pro-inflammatory cytokines (IL-6 and TNF-α) • Reduced the levels of LPS in plasma and brain • Increased expression of TJ proteins (claudin-5, occludin, and ZO-1) and AJ protein (VE-cadherin) in brain tissues • Inhibited the TLR4- and RIG-I-mediated NF-κB signaling pathway • Reduced microglial and astrocytic activation in the hippocampus • Protected against aging-associated cognitive impairments	^758^
* Lactobacillus plantarum* MA2	D-galactose/AlCl_3_-induced AD rats	Daily treatment for 12 weeks	• Improved intestinal mucosal barrier integrity • Increased expression of TJ protein (occludin) in the intestine and brain tissues • Reduced neuronal degeneration and Aβ accumulation in the hippocampus and cortex • Inhibited microglial activation and TLR4/MYD88/NLRP3 signaling • Improved cognitive impairments and anxiety-like behaviors	^759^
Parkinson’s disease				
* Lactobacillus plantarum* (CCFM405)	4-month-old rotenone-induced PD mice	Daily treatment for 8 weeks	• Increased fecal and serum BCAAs (valine, isoleucine, leucine) • Restored goblet cell number • Increased expression of TJ proteins (ZO-1 and occludin) in the colon • Reduced the levels of pro-inflammatory cytokines in the colon and midbrain • Alleviated constipation symptoms and colon shortening • Inhibited microglial and astrocytic activation in SN • Reduced the loss of dopaminergic neurons in striatum • Restored the levels of neurotransmitters in striatum • Alleviated rotenone-induced motor dysfunction	^456^
* Bifidobacterium breve* (CCFM1067)	3-month-old MPTP-induced PD mice	Daily treatment for 33 days	• Increased the levels of fecal SCFAs (acetic acid and butyric acid) • Increased expression of TJ proteins (ZO-1, occludin, and claudin-1) in the colon and striatum • Reduced pro-inflammatory cytokines and increased anti-inflammatory cytokine in the colon and striatum • Increased the levels of neurotrophic factors (BDNF and GDNF) in striatum • Inhibited microglial and astrocytic activation in striatum • Restored the levels of neurotransmitters • Alleviated MPTP-induced motor dysfunction	^260^
* Lactococcus lactis* MG1363-pMG36e-GLP-1	MPTP- induced PD mice	Daily treatment for 7 days	• Increased the levels of GLP-1 and GLP-1 receptor in SN • Increased expression of TJ proteins (ZO-1 and occludin) in colon and SN • Inhibited ferroptosis in SN by activating the Keap1-Nrf2-GPX4 pathway • Reduced α-synuclein aggregation in SN • Reduced the loss of dopaminergic neurons in SN • Alleviated MPTP-induced motor dysfunction	^617^
Probiotic cocktail (*Lactobacillus rhamnosus GG, Bifidobacterium animalis lactis*, and *Lactobacillus acidophilus*)	3-month-old MPTP-induced PD mice	Daily treatment for 30 days	• Increased the levels of neurotrophic factors (BDNF and GDNF) in SN • Inhibited microglial and astrocytic activation in SN • Reduced the loss of dopaminergic neurons in SN and striatum • Alleviated MPTP- and rotenone-induced motor dysfunction	^321^
	3-month-old rotenone-induced PD mice			
* Clostridium butyricum*	3-month-old MPTP-induced PD mice	Daily treatment for 4 weeks	• Increased the levels of colonic GLP-1 and cerebral GLP-1 receptor • Increased the levels of colonic GPR41/43 • Inhibited microglial activation and synaptic dysfunction in SN • Reduced the loss of dopaminergic neurons in SN • Alleviated MPTP-induced motor dysfunction	^316^
* Probiotic suspension* Symprove*™*	DSP-4 and 6-OHDA-induced PD mice	Daily treatment for 24 days	• Increased the levels of fecal butyrate • Increased expression of TJ protein (occludin) in the intestine • Reduced plasma levels of LPS and pro-inflammatory cytokines (TNF-α. IL-1β, and IL-6) • Reduced microglial and astrocytic activation in striatum	^760^
* Lactobacillus plantarum* PS128	10-week-old MPTP-induced PD mice	Daily treatment for 4 weeks	• Increased the levels of neurotrophic factors (mature BDNF and NGF) in striatum • Increased the levels of neurotransmitters in striatum • Inhibited microglial and astrocytic activation in striatum • Reduced oxidative stress in the striatum and midbrain • Reduced the expression of pro-inflammatory cytokines (TNF-α. IL-1β, and IL-6) in striatum and midbrain • Reduced the loss of dopaminergic neurons in SN and striatum • Alleviated MPTP-induced motor dysfunction	^492^
Amyotrophic lateral sclerosis				
* Lacticaseibacillus rhamnosus* HA-114	*C. elegans* ALS strain (TDP-43^A315T^ and FUS^S57Δ)^	NGM plates streaked with probiotic as food source	• Rescued paralysis phenotypes and neurodegeneration • Exerted neuroprotection via fatty acid metabolism (mitochondrial β-oxidation) • Restored lipid homeostasis and reduced lipid accumulation • HA-114 fatty acids extracts, but not protein extracts, rescued age-dependent paralysis phenotype in FUS^S57Δ^ worms	^761^
Huntington’s disease				
* Lacticaseibacillus rhamnosus* HA-114	*C. elegans* strains expressing pan-neuronal polyglutamine repeats (Q40 and Q67)	NGM plates streaked with probiotic as food source	• Rescued paralysis phenotypes • Restored lipid homeostasis and reduced lipid accumulation	^761^

**Table 2:** In vivo evidences of prebiotics in neurodegenerative disease models

Types of prebiotics	Subjects	Intervention duration	Key findings	References
Alzheimer’s disease				
Mannan oligosaccharide (MOS)	8-month-old male 5xFAD mice	8 weeks	• Increased the relative abundance of butyrate-producing bacteria and increased the level of butyrate in feces and serum • Attenuated the loss of goblet cells and shrunk crypts in the colon • Improved intestinal barrier integrity • Reduced serum LPS level • Reduced microglial activation and Aβ deposition in prefrontal cortex, hippocampus and amygdala • Reduced mRNA expressions of amyloid precursor protein (APP) and β-secretase (Bace1) in the cortex and hippocampus • Reduced oxidative stress in the brain • Attenuated cognitive deficits and anxiety-like behaviors	^304^
Xylooligosaccharides (XOS)	Surgery-induced cognitive dysfunction in APP/PS1 mice	Daily treatment for 5 weeks	• Restored the integrity of intestinal barrier and BBB by increasing the expression of TJ proteins (ZO-1 and occludin) in intestine and hippocampus • Reduced the expression of pro-inflammatory cytokines (IL-1β and IL-6) and immunosuppressive cytokine (IL-10) in colon and hippocampus • Attenuated surgery-induced microglial activation and reduction in TREM2 expression • Attenuated surgery-induced spatial memory deficits	^762^
* Polygonatum sibiricum* polysaccharides (PSP-1)	6-month-old 5xFAD mice	3 months	• Increased the mRNA levels of TJ proteins (ZO-1 and occludin) and number of goblet cells in the ileum • Reduced intestinal Aβ deposition • Prevented synaptic loss and reduced Aβ deposition in hippocampus and cortex • Enhanced microglial phagocytosis and clearance of Aβ • Attenuated spatial memory deficits	^763^
β-Glucan	APP/PS1 mice	β-Glucan : Daily treatment for 1 month	• Increased the levels of SCFAs (propionate, butyrate, and valerate) in colon • Stimulated the proliferation of intestinal wall cells and the restoration of intestinal microecology • Reduced microglial and astrocytic activation in hippocampus • Reduced the expression of pro-inflammatory cytokines (IL-6 and IL-1β), NF-κB and NLRP3 in hippocampus and cerebral cortex • Reduced Aβ deposition in hippocampus and cerebral cortex • Attenuated cognitive impairment • FMT from β-glucan-treated mice attenuated cognitive impairment, reduced Aβ deposition and neuroinflammation	^305^
		FMT: Daily administration for 2 months		
R13 (prodrug of 7,8-dihydroxyflavone)	6-month-old 5xFAD mice	Daily treatment for 3 months	• Inhibited C/EBPβ/AEP signaling in the gut, reduced gut leakage and reduced the levels of IL-6 in the gut • Reduced APP and Tau proteolytic fragmentation by the AEP and inhibited Aβ pathology in the gut • Reduced oxidative stress in gut and brain	^204^
* Schisandra chinensis* polysaccharide (SCP-2)	Aβ_25–35_-induced AD rats	2 months	• Restored the levels of fecal SCFAs (acetate, isobutyrate, valerate, and isovaleric acid) • Increased expression of TJ proteins (ZO-1 and occludin) in intestine • Reduced serum levels of pro-inflammatory cytokines (IL-6, TNF-α, and IL-1β) • Reduced microglial activation • Attenuated learning and memory deficits	^764^
* Dendrobium officinale* polysaccharide (DOP)	Male C57BL/6 J mice with circadian rhythm disruption	4 weeks	• Increased expression of TJ proteins (ZO-1 and occludin) in intestine • Reduced serum levels of LPS • Reduced the hippocampal expression of pro-inflammatory cytokines (TNF-α, IL-1β, and IL-6) • Increased the hippocampal expression of anti-inflammatory cytokines (IL-10 and IL-4) • Reduced hippocampal Aβ deposition and neuronal damage • Prevented deficits in recognition and spatial memory	^765^
* Sparassis crispa*-1 polysaccharide (SCP-1)	D-galactose/AlCl3-induced C57BL/6J male mice	Daily treatment for 4 weeks	• Restored the levels of fecal SCFAs (acetate, propionate, and butyrate) • Increased expression of TJ proteins (occludin and ZO-1) in the intestine • Reduced serum levels of LPS • Reduced the expression of pro-inflammatory cytokines (IL-6, IL-1β and TNF-α) in the serum and brain • Reduced Aβ deposition in hippocampus • Inhibited microglial and astrocytic activation in hippocampus • Restored the levels of inhibitory neurotransmitters (GABA and acetylcholine) and reduced the levels of excitatory neurotransmitter (glutamate) • Attenuated chemically-induced cognitive impairment	^479^
Parkinson’s disease				
Polymannuronic acid (brown seaweed polysaccharide)	13-week-old MPTP-induced PD mice	Daily treatment for 4 weeks	• Increased the levels of fecal SCFAs (acetate, propionate, butyrate) • Increased expression of TJ proteins (ZO-1 and occludin) in colon and SN • Reduced the expression of pro-inflammatory cytokines (TNF-α and IL-6) in serum, colon and SN • Restored the levels of neurotransmitters in striatum • Reduced the loss of dopaminergic neurons in SN and cortex • Alleviated MPTP-induced motor dysfunction	^490^
Prebiotic high-fiber diets	22-week-old ASO mice	17 weeks	• Increased the levels of fecal SCFAs (acetate, propionate, butyrate, isobutyrate) • Inhibited microglial activation in SN and striatum • Induced neuroprotective microglial phenotype (TREM2 upregulation) • Reduced α-synuclein aggregation in the SN • Alleviated motor dysfunction	^318^
Polymannuronic acid and *Lacticaseibacillus rhamnosus* GG (Synbiotic)	3-month-old MPTP-induced PD mice	Daily treatment for 5 weeks	Compared with prebiotic and probiotic alone, synbiotic: • Increased expression of TJ proteins (ZO-1 and occludin) in striatum • Increased the levels of neurotrophic factors (BDNF and GDNF) in striatum • Reduced apoptosis in striatum • Reduced the loss of dopaminergic neurons in midbrain and striatum • Alleviated MPTP-induced motor dysfunction	^526^
Amyotrophic lateral sclerosis				
Galacto-oligosaccharide (GOS) and GOS-rich prebiotic yogurt	4-month-old SOD1^G93A^ mice	Daily treatment for 74 days	• Significant delay of disease onset and prolonged lifespan • Increased the levels of serum folate and vitamin B12 • Reduced the levels of serum homocysteine • Reduced motor neuron death • Reduced muscle atrophy by reducing oxidative stress • Inhibited microglial and astrocytic activation in the spinal cord • Reduced the expression of pro-inflammatory molecules (iNOS and TNF-α) • Inhibited apoptosis in spinal cord	^766^

In addition, these studies have reported an increased relative abundance of beneficial gut microbes and a reduced relative abundance of harmful gut microbes. However, concerns have been raised regarding the limitations and risk of bias associated with the use of relative abundance due to technical variation, which may lead to erroneous interpretations founded on proportional profiling.^717,718^ The changes in the relative abundance of a microbial taxon are inherently compensated by equivalent increases or decreases in the remaining taxa, resulting in correlation biases that adversely affect downstream analysis.^719^ Bias can be introduced throughout all stages of sample processing, including the selection of sample collection protocol, preservative choice, storage temperature, DNA extraction protocol, library preparation, sequencing platform, and bioinformatics analysis pipeline.^720–723^ Thus, it is recommended to harmonize study protocols and incorporate quantitative microbiome profiling (absolute quantification) to complement relative abundance data.^718,719,722^ This approach will provide a more comprehensive understanding of the impact of microbiome-based therapeutics on the gut microbiome and neurodegenerative disease pathologies.

### Fecal microbiota transplantation (FMT)

FMT refers to the procedure of transferring a healthy donor’s microbial ecosystem to the gastrointestinal tract of a recipient, with the aim of modifying the recipient’s gut microbiome and treating diseases associated with gut dysbiosis.^724,725^ It is considered a broad and largely untargeted gut microbiota modulation strategy, pitting donor microbial ecosystems against those of the recipient.^726^ However, FMT has undergone significant advancements over the years, evolving from a relatively crude procedure of transferring fresh donor stool to a mainstream treatment option. This evolution is enabled by the development of standardized FMT products, with increased emphasis on their pharmaceutical formulation, pharmacokinetics, pharmacodynamics, and toxicity.^727^ A key strength of FMT is the ability to transfer both the favorable microbes, as well as their intricate supporting ecosystem.^726^ FMT is most commonly applied in the management of *Clostridioides difficile* infection and inflammatory bowel disease.^728^ Notably, FMT has achieved a significant milestone following the recent FDA approval of RBX2660 (Rebyota) and SER-109 (Vowst) for the prevention of recurrent *Clostridioides difficile* infection, highlighting the therapeutic potential of FMT in human disease.^729–731^ Interestingly, SER-109 is an oral microbiome therapeutic administered in the capsule dosage form, eliminating the need for endoscopy procedures.^729^

As discussed previously, the development of neurodegenerative diseases and glial activation are linked to gut dysbiosis and altered microbial metabolites. In this section, we summarize the findings of in vivo studies investigating the applications of FMT in neurodegenerative diseases (Table 3). In general, FMT from either healthy control mice or WT mice has demonstrated promising results in correcting gut dysbiosis and alleviating neurodegeneration in recipient mouse models of neurodegenerative diseases. These positive outcomes can be ascribed to the promotion of beneficial microbes and the elevation of beneficial metabolites, resulting in the restoration of intestinal barrier and BBB integrity. Consequently, the recipient mice exhibited a reduction in systemic inflammation, glial activation, and neuroinflammation.

**Table 3:** In vivo evidence of FMT in neurodegenerative disease models

FMT donor	Subjects	Intervention duration	Key findings	References
Alzheimer’s disease				
WT mice	7-month-old APP/PS1 mice	Antibiotics pre-treatment for 3 days, followed by daily FMT for 4 weeks	- Reversed the alterations in gut microbiota composition - Increased the level of fecal SCFA (butyrate) - Reduced Aβ deposition and tau hyperphosphorylation - Inhibited neuroinflammation by reducing the levels of COX-2 and CD11b in cortex and hippocampus - Attenuated synaptic dysfunction in cortex and hippocampus - Attenuated cognitive impairment	^767^
WT mice	6-month-old ADLP^APT^ mice	Administration 5 days/week for 4 months	- Restored the gut microbiota composition resembling WT mice after 4 months of FMT - Restored the levels of circulating Ly6C^+^ inflammatory monocytes to WT levels - Reduced Aβ deposition in frontal cortex and tau hyperphosphorylation in the hippocampus - Inhibited microglial and astrocytic activation in frontal cortex - Attenuated cognitive impairment	^635^
	4.5-month-old ADLP^APT^ mice	Antibiotic pre-treatment for 4 weeks, followed by FMT for 4 weeks	- Reduced Aβ deposition in frontal cortex and tau hyperphosphorylation in hippocampus - Inhibited microglial and astrocytic activation in frontal cortex - Attenuated behavioral impairment	
Parkinson’s disease				
Healthy control mice	10-week-old MPTP-induced PD mice	Daily administration for 7 days	- Reversed the MPTP-induced gut dysbiosis - Inhibited MPTP-induced elevation in fecal SCFAs - Inhibited TLR4/TBK1/NF-κB/TNF-α signaling pathway in colon and striatum - Restored the levels of neurotransmitters in striatum - Inhibited microglial and astrocytic activation in SN - Reduced the loss of dopaminergic neurons in SN - Alleviated MPTP-induced motor dysfunction	^217^
Healthy human controls	3-month-old MPTP-induced PD mice	Daily administration for 10 days	- Reversed the MPTP-induced gut dysbiosis - Increased expression of TJ protein (ZO-1) in the colon - Reduced the expression of pro-inflammatory cytokine (IL-1β) in colon - Activates the AMPK/SOD2 signaling pathway in colon and SN - Attenuated MPTP-induced BBB damage by improving the survival of pericytes in SN and striatum - Inhibited microglial and astrocytic activation in SN and striatum - Reduced the loss of dopaminergic neurons in SN and striatum - Alleviated MPTP-induced motor dysfunction	^261^
Healthy control mice	10-week-old MPTP-induced PD mice	Daily administration for 7 days	- Inhibited MPTP-induced elevation in fecal SCFAs - Inhibited TLR4/PI3K/AKT/NF-κB signaling pathway in SN and striatum - Inhibited microglial activation in SN - Reduced the loss of dopaminergic neurons in SN - Reduced α-synuclein aggregation in the SN - Alleviated MPTP-induced motor dysfunction	^333^
Healthy control mice	15-week-old rotenone-induced PD mice	Daily administration for 2 weeks	- Reversed the rotenone-induced gut dysbiosis - Increased expression of TJ proteins (ZO-1, occludin, and claudin-1) in the colon - Increased expression of TJ proteins (ZO-1, occludin, and claudin-5) in SN - Alleviated rotenone-induced GI dysfunction - Reduced serum levels of LPS, LBP and pro-inflammatory cytokines (IL-6, IL-1β, and TNF-α) - Inhibited TLR4/MyD88/NF-κB signaling pathway in colon and SN - Inhibited microglial and astrocytic activation in SN - Reduced the loss of dopaminergic neurons in SN - Reduced α-synuclein aggregation in the SN - Alleviated rotenone-induced motor dysfunction	^525^
Amyotrophic lateral sclerosis				
Age-matched (13-week-old) *C9orf72* mice housed at Harvard Institute (pro-inflammatory) or Broad Institute (pro-survival)	*C9orf72*^+/+^ and *C9orf72*^−/−^ mice reared at Harvard Institute	Antibiotic pre-treatment twice daily for 2 weeks, followed by FMT once daily for 2 days	- Transplantation of pro-survival gut microbiota significantly improved the inflammatory and autoimmune phenotypes (reduced plasma anti-dsDNA antibody activity, reduced blood neutrophil count and increased blood platelet count) - Transplantation of pro-inflammatory gut microbiota did not improve these measures	^57^
Huntington’s disease				
WT mice	20-week-old R6/1 mice	Antibiotic pre-treatment twice daily for 7 days, followed by FMT for 3 days with 2 days of spacing	- Inefficient FMT engraftment in male R6/1 mice - Rescued cognitive impairment in female R6/1 mice - No improvement in intestinal dysfunction and motor dysfunction	^244^

Interestingly, the rearing environment of FMT donors may influence the outcomes of FMT. A recent study showed that FMT from *C9orf72*-mutant mice housed at pro-survival environment (Broad Institute) effectively ameliorated the autoimmune inflammatory phenotypes in recipient *C9orf72*-mutant mice, whereas FMT from those housed at pro-inflammatory environment (Harvard Institute) did not improve these phenotypes.^57^ In addition, the extent of FMT engraftment in recipient mice may be influenced by the gender of the recipients. This phenomenon is exemplified in the R6/1 HD mouse model, wherein male R6/1 mice exhibited greater resistance to FMT engraftment when compared to female R6/1 mice. Consequently, the cognitive function of male R6/1 mice showed no discernible improvement as compared to their female counterparts.^244^ Similar sexual dimorphism of FMT has been reported in a clinical trial of irritable bowel syndrome, wherein female patients exhibit significantly better responses compared to male patients.^732^ Thus, future studies should account for these factors, given their substantial influence on the efficacy and response to FMT.

### Clinical applications of microbiome-based therapeutics

Given the accumulating evidence demonstrating the potential of gut microbiota manipulations on cognitive functions, intense interest exists in applying microbiome-based therapeutics to alleviate the progression of neurodegenerative diseases. In this section, we summarize the completed and ongoing clinical trials in Tables 4 and 5, respectively. A recent study has identified constipation/bowel movement frequency as a potential mediator in the relationship between gut microbiota and the likelihood of developing prodromal PD.^52^ Thus, we have included clinical trials that investigated the effects on constipation as an endpoint.

**Table 4:** Completed clinical trials targeting microbiota–gut–brain axis in neurodegenerative diseases

Intervention tested	Disease	Participants	Dosage	Control	Duration	Study design	Phase	Main findings	Clinical trial identifier	Ref.
Probiotics										
Probiotic capsules containing: - *Bifidobacterium breve* A1 (MCC1274)	MCI	Individuals with MCI *N* = 40 (Control) *N* = 40 (Intervention)	2 × 10^10^ CFU/day	Placebo capsules containing maize starch	16 weeks	Double-blind, placebo-controlled RCT	N/A	- Improved cognitive function (RBNAS total score, immediate memory, visuospatial/constructional score and delayed memory)	UMIN000037725	^768^
Probiotic capsules containing: *- Bifidobacterium breve* A1	MCI	Individuals with MCI *N* = 58 (Control) *N* = 59 (Intervention)	2 × 10^10^ CFU/day	Placebo capsules containing cornstarch	12 weeks	Double-blind, placebo-controlled RCT	N/A	- Probiotics improved cognitive function (RBANS total score and delayed memory), but no significant difference between probiotics and placebo	UMIN000031679	^769^
* Lactobacillus Plantarum* C29-Fermented Soybean (DW2009)	MCI	Individuals with MCI *N* = 50 (Control) *N* = 50 (Intervention	1 × 10^10^ CFU/day	Placebo capsules containing cellulose	12 weeks	Double-blind, placebo-controlled RCT	N/A	- Improved cognitive function (attention function) - Increased serum BDNF levels	KCT0002346	^770^
Probiotic milk containing: - *Lactobacillus acidophilus* - *Lactobacillus casei* - *Bifidobacterium bifidum* - *Lactobacillus fermentum*	AD	AD patients *N* = 30 (Control) *N* = 30 (Intervention)	2 × 10^9^ CFU/day for each strain	Placebo milk	12 weeks	Double-blind, placebo-controlled RCT	Phase II	- Improved MMSE scores - Reduced levels of serum hs-CRP (inflammation) and plasma malondialdehyde (oxidative stress) - Improved markers of insulin metabolism and triglycerides levels - No considerable effect on other biomarkers of inflammation and oxidative stress	IRCT201511305623N60	^771^
Probiotic capsule containing: - *Lactobacillus rhamnosus* or - *Bifidobacterium longum* R0175	AD	AD patients *N* = 30 (Control) *N* = 30 (*L. rhamnosus* intervention) *N* = 30 (*B. longum* R0175 intervention)	2 × 10^15^ CFU twice daily	Placebo capsules containing xylitol, maltodextrin, and malic acid twice daily	12 weeks	Double-blind, placebo-controlled RCT	Phase III	- Improved MMSE and CFT scores in both intervention groups - Improved IADL and GAD-7 scales in both intervention groups	IRCT20210513051277N1	^772^
200 μg selenium with/without probiotic supplements containing: - *Lactobacillus acidophilus* - *Bifidobacterium bifidum* - *Bifidobacterium longum*	AD	AD patients *N* = 26 (Control) *N* = 26 (Selenium only) *N* = 27 (Intervention + Selenium)	2 × 10^9^ CFU/day for each strain	Starch	12 weeks	Double-blind, placebo-controlled RCT	Phase II	Selenium with probiotics: - Improved MMSE scores - Improved markers of insulin metabolism - Reduced serum hs-CRP, triglycerides, VLDL-cholesterol, LDL-cholesterol, total-/HDL-cholesterol and insulin - Increased serum TAC and GSH levels	IRCT20170612034497N5	^773^
Probiotic milk with fructo-oligosaccharide and lactose - *Lactobacillus acidophilus* - *Lactobacillus casei* - *Lactobacillus lactis* - *Bifidobacterium infantis* - *Bifidobacterium longum*	PD	PD patients *N* = 26 (Control) *N* = 22 (Intervention)	30 × 10^9^ CFU twice daily	Granulated milk containing lactose	8 weeks	Triple-blind, placebo-controlled RCT	Phase III	- Improved constipation-related parameters (weekly bowel movement, bowel opening frequency, gut transit time) - No significant differences in MDS-UPDRS II and III scores, NMSS, and PDQ-39SI scores	NCT04451096	^774^
Probiotic capsules containing: - *Lactobacillus acidophilus* - *Bifidobacterium bifidum* - *Lactobacillus reuteri* - *Lactobacillus fermentum*	PD	PD patients *N* = 30 (Control) *N* = 30 (Intervention)	2 × 10^9^ CFU/day for each strain	Placebo capsules	12 weeks	Double-blind, placebo-controlled RCT	Phase II	- Improved MDS-UPDRS scores - Reduced the levels of hs-CRP and malondialdehyde - Increased the level of GSH - Improved biomarkers of insulin metabolism	IRCT2017082434497N4	^775^
Conventional regimen (Benserazide and dopamine agonist) with or without probiotic powder containing: *Bifidobacterium animalis subsp. lactis* Probio-M8 (Probio-M8)	PD	PD patients *N* = 34 (Control) *N* = 48 (Intervention)	3 × 10^10^ CFU/day	Conventional regimen with placebo powder (maltodextrin)	12 weeks	Double-blind, placebo-controlled RCT	N/A	- Improved UPDRS-III and MMSE scores - Improved severity of depression (HAMD-17 scores) - Improved GI-related parameters (constipation) - Increased the diversity of species-level genome bins involved in tryptophan degradation, as well as SCFAs, GABA, and secondary BAs biosynthesis - Increased the serum levels of acetic acid and dopamine - Reduced the serum levels of tryptophan and glutamine	ChiCTR1800016977	^491^
* Lacticaseibacillus paracasei* strain Shirota fermented milk	PD	PD patients *N* = 63 (Control) *N* = 65 (Intervention)	1 × 10^10^ CFU/day at breakfast	Placebo milk	12 weeks	Double-blind, placebo-controlled RCT	N/A	- Improved constipation-related parameters (Wexner score, BSFS, bowel movement, reduction in use of laxatives, PACQoL) - Improved non-motor symptoms (NMSS, HAMD-17, and HAMA) - Improved quality of life (PDQ-39 scores) - Improved MDS-UPDRS I score (non-motor experiences of daily living) - No significant effect on other MDS-UPDRS scores	ChiCTR1800016795	^776^
Probiotic capsule containing: - *Lactobacillus fermentum* - *Lactobacillus plantarum* - *Bifidobacterium lactis* or - *Lactobacillus acidophilus* - *Bifidobacterium bifidum* - *Bifidobacterium longum*	AD	AD patients *N* = 30 (Control) *N* = 30 (Intervention)	3 × 10^9^ CFU per capsule; one capsule of each type every other day	Placebo capsules containing 500 mg maltodextrine	12 weeks	Double-blind, placebo-controlled RCT	N/A	- No significant improvement in TYM cognitive test - No significant effects on biomarkers of inflammation and oxidative stress	IRCT2017061534549N1	^777^
Probiotic sachets containing: - *Bifidobacterium breve* A1 (MCC1274)	AD	AD patients *N* = 60 (Control) *N* = 55 (Intervention)	2 × 10^10^ CFU/day	Placebo sachets containing maize starch	24 weeks	Double-blind, placebo-controlled RCT	N/A	- No significant difference in ADAS-Jcog total score between probiotic and placebo, except orientation subscore - No significant difference in MMSE total score between probiotic and placebo - Suppressed brain atrophy progression - No marked changes in the overall composition of the gut microbiota	UMIN000031507	^778^
Probiotic capsules containing: - *Lactobacillus acidophilus* - *Bifidobacterium bifidum* - *Lactobacillus reuteri* - *Lactobacillus fermentum*	PD	PD patients *N* = 25 (Control) *N* = 25 (Intervention)	2 × 10^9^ CFU/day for each strain	Placebo capsules	12 weeks	Double-blind, placebo-controlled RCT	N/A	- Downregulated the expression of pro-inflammatory cytokines (IL-1, IL-8, and TNF-α) in PBMC - Upregulated the expression of TGF-β and PPAR-γ in PBMC - No effects on the expression of LDLR and VEGF in PBMC - No effects on nitric oxide and GSH	IRCT20170513033941N34	^779^
Fermented milk containing prebiotic fiber and multiple probiotics strains: - *Streptococcus salivarius subsp thermophilus* - *Enterococcus faecium* - *Lactobacillus rhamnosus* GG - *Lactobacillus acidophilus* - *Lactobacillus plantarum* - *Lactobacillus paracasei* - *Lactobacillus delbrueckii subsp. bulgaricus* - *Bifidobacterium* *Breve* - *Bifidobacterium animalis subsp. lactis*	PD	PD patients *N* = 40 (Control) *N* = 80 (Intervention)	2.5 × 10^11^ CFU/day at breakfast	placebo milk (pasteurized, fermented, fiber-free milk)	4 weeks	Double-blind, placebo-controlled RCT	N/A	- Improved constipation-related parameters (complete bowel movement, stool frequency, stool consistency, reduction in use of laxatives)	NCT02459717	^780^
Probiotics capsules containing: - *Lactobacillus acidophilus* - *Lactobacillus reuteri* - *Lactobacillus gasseri* - *Lactobacillus rhamnosus* - *Bifidobacterium bifidum* - *Bifidobacterium longum* - *Enterococcus faecalis* - *Enterococcus faecium*	PD	PD patients *N* = 38 (Control) *N* = 34 (Intervention)	1 × 10^10^ CFU/day for each strain	Placebo capsules (maltodextrin)	4 weeks	Double-blind, placebo-controlled RCT	N/A	- Improved constipation-related parameters (spontaneous bowel movements, stool consistency, quality of life related to constipation) - No significant change in fecal calprotectin	NCT03377322	^781^
Probiotic capsules containing: - *Lactobacillus plantarum* PS128	PD	PD patients *N* = 25 (Intervention)	6 × 10^10^ CFU/day	N/A	12 weeks	Open-label, single-arm	N/A	- Improved UPDRS motor scores, akinesia and rigidity subscores in OFF state - Improved UPDRS motor scores and total UPDRS scores in ON state - Reduced the duration of OFF period - Increased the duration of ON period - Improved quality of life (PDQ-39 scores) - No significant improvement in non-motor symptoms - Reduced the levels of plasma myeloperoxidase and urine creatinine	NCT04389762	^782^
Probiotic supplement containing: - *Streptococcus thermophilus* - *Lactobacillus fermentum* - *Lactobacillus delbrueckii subsp. delbrueckii* - *Lactobacillus plantarum* - *Lactobacillus salivarius*	ALS	ALS patients *N* = 25 (Control) *N* = 25 (Intervention)	Daily dosage: *S. thermophilus* (5 × 10^9^ CFU) *L. fermentum* (4 × 10^9^ CFU) *L. delbrueckii subsp. delbrueckii*, *L. plantarum* & *L. salivarius* (2 × 10^9^ CFU for each strain)	Placebo for 3 months, followed by probiotic for 3 months	6 months	Double-blind, placebo-controlled RCT	Phase I	- No substantial alterations in the gut microbial composition - No influence on disease progression (ALSFRS-R score) - No improvement in pulmonary function test	CE 107/14	^783^
Prebiotics										
Prebiotic bar (10 g fiber) containing: -30% resistant starch (raw potato starch) -30% resistant maltodextrin (Nutriose^TM^) -30% stabilized rice bran -10% agave branched inulin	PD	*N* = 10 (newly diagnosed, non-medicated PD participants *N* = 10 (treated PD participants	1 bar daily for 3 days, followed by 1 bar twice daily for 7 days	N/A	10 days	Open-label, non-randomized study	N/A	- Increased relative abundance of putative SCFA-producing species - Reduced relative abundance of pro-inflammatory phylum Proteobacteria and species *Escherichia coli* - Increased plasma SCFAs - Reduced plasma zonulin (marker of intestinal barrier dysfunction and inflammation) - Reduced calprotectin (intestinal inflammation) - Reduced plasma neurofilament (marker of neurodegeneration) - No change in LBP, serum cytokines and CRP, HMGB-1 and BDNF - Improved total UPDRS scores - Improved GI symptoms	NCT04512599	^332^
Synbiotics (probiotic + prebiotic)										
Synbiotic capsules containing: - *Lactobacillus rhamnosus* GG - prebiotic inulin (200 mg)	MCI	Individuals with MCI *N* = 83 (Control) *N* = 86 (Intervention)	1 × 10^10^ CFU/day	Placebo capsules containing microcrystalline cellulose	12 weeks	Double-blind, placebo-controlled RCT	N/A	- Higher relative abundance of the genus *Prevotella* in MCI group than cognitively intact individuals - Probiotic reduced the relative abundance of the genus *Prevotella* and *Dehalobacterium* in MCI group, but not in cognitively intact individuals	NCT03080818	^784^
Synbiotic sachets containing: - *Lactobacillus acidophilus* - *Lactobacillus rhamnosus* - *Lactobacillus plantarum* - *Bifidobacterium longum* - *Streptococcus thermophilus* - prebiotic inulin (4 g)	PD	PD patients *N* = 40 (Control) *N* = 40 (Intervention)	5 × 10^9^ CFU/day	Placebo sachets containing maltodextrin	12 weeks	Double-blind, placebo-controlled RCT	N/A	- Improved oxidative stress biomarkers (increased serum TAC, reduced serum malondialdehyde and OSI) - No significant difference in serum TOS and GSH - Improved well-being and cognitive impairment domains of PDQ-39 - Improved depressive symptoms (BDI-II and HADS-DEP scores)	IRCT20180818040827N2	^785^
Fecal microbiota transplantation										
Orally administered FMT capsules (donor: healthy adults)	MCI, AD & FTD	2 MCI patients, 2 AD patients and 1 FTD patient *N* = 5 (Intervention)	40 capsules per intake, administered every other week	N/A	6 months	Open-label, single-arm trial	N/A	- Slight improvement in cognitive function in MCI patients (ADL and ADAS-Cog scores) - No improvement in cognitive function in AD and FTD patients - No adverse effects	CHiCTR2100043548	^786^
Orally administered lyophilized FMT capsules	PD	PD patients *N* = 4 (Control) *N* = 8 (Intervention)	1 capsule twice weekly for 12 weeks (total 24 capsules)	Placebo capsules	12 months	Double-blind, placebo-controlled RCT	Phase I	- Improved UPDRS Motor and UPDRS-Total scores - Subjective improvement in constipation, falls, sleep impairment, motor deficits and global Parkinson’s symptoms - Improved constipation-related parameters (gut transit time, motility index) - Increased proportion of phylum Firmicutes - Reduced proportion of Proteobacteria - No significant improvement in geriatric depression score, Parkinson’s Anxiety Score, non-motor symptoms and PDQ-39 score - Adverse effects: Non-severe transient upper GI symptoms	NCT03671785	^787^
FMT from two healthy donors, administered via colonoscopy in 3 portions: -Terminal ileum (100 mL) -Cecum (100 mL) -Along the rest of colon (100 mL)	PD	PD patients *N* = 6 (Intervention)	One administration at the beginning of study	N/A	6 months	Single-arm case series	Phase II & III	- Improved UPDRS-III and NMSS scores - Improved constipation-related parameters (BSFS and Wexner score) - 1 patient experienced vasovagal pre-syncope 24 h post-FMT	NCT03876327	^788^
Frozen fecal microbiota was obtained from the China fmtBank, and the suspension is transplanted into patients’ intestine through a nasoduodenal tube	PD	PD patients *N* = 13 (Control) *N* = 11 (Intervention)	One administration at the beginning of study	N/A	12 weeks	Non-randomized controlled trial	N/A	- Improved Hoehn-Yahr grade, UPDRS, and NMSS scores - Improved constipation-related parameters (Wexner and PACQoL scores, gut transit time, and Lactulose H_2_ Breath Test) - Adverse effects: Mild GI symptoms and venting	ChiCTR2000040891	^789^

*AD* Alzheimer’s disease, *PD* Parkinson’s disease, *ALS* amyotrophic lateral sclerosis, *RBANS* repeatable battery for the assessment of neuropsychological status, *BDNF* brain-derived neurotrophic factor, *MMSE* mini-mental state examination, *hs-CRP* high sensitivity C-reactive protein, *CFT* categorical verbal fluency test, *IADL* instrumental activities of daily living, *GAD-7* generalized anxiety disorder, *VLDL-cholesterol* very low-density lipoprotein-cholesterol, *LDL-cholesterol* low-density lipoprotein-cholesterol, *HDL-cholesterol* high-density lipoprotein-cholesterol, *TAC* total antioxidant capacity, *GSH* total glutathione, *UPDRS* unified Parkinson’s disease rating scale, *MDS-UPDRS* movement disorder society-sponsored revision of the unified Parkinson’s disease rating scale, *NMSS* non-motor symptoms scale, *PDQ-39* 39-item Parkinson’s Disease Questionnaire, *HAMD-17* Hamilton Depression Scale-17, *SCFA* short-chain fatty acids, *GABA* γ-aminobutyric acid, *BSFS* Bristol Stool Form Scale, *PACQoL* Patient Assessment of Constipation Quality Of Life, *HAMA* Hamilton Anxiety Scale, *TYM* test your memory, *ADAS-Cog* Alzheimer’s Disease Assessment Scale–Cognitive subscale, *PPAR* peroxisome proliferators-activated receptor, *LDLR* low-density lipoprotein receptor, *PBMC* peripheral blood mononuclear cells, *TGF-β* transforming growth factor beta, *VEGF* vascular endothelial growth factor, *ALSFRS-R* ALS Functional Rating Scale–Revised, *LBP* lipopolysaccharide-binding protein, *HMGB-1* high-mobility group box 1 protein, *OSI* oxidative stress index, *TOS* total oxidant capacity, *BDI-II* Beck’s Depression Inventory-II, *HADS-DEP* Hospital Anxiety and Depression Scale - Depression subscale, *ADL* activities of daily living, *N/A* data not applicable

**Table 5:** Ongoing clinical trials targeting microbiota–gut–brain axis in neurodegenerative diseases

Intervention tested	Disease	Control	Duration	Study design	Phase	Status	NCT number
Probiotics							
Probiotic containing Bifidobacterium	Amnestic MCI	Placebo	3 months	Triple-blind, placebo-controlled RCT (*N* = 90)	N/A	Completion date Mar 2022 No results posted	NCT03991195
Normal dose probiotic powder containing: - *Bifidobacterium breve* Bv-889 - *Bifidobacterium longum subsp. infantis* BLI-02 - *Bifidobacterium bifidum* VDD088 - *Bifidobacterium* animalis subsp. lactis CP-9 - *Lactobacillus plantarum* PL-0	AD	Low-dose probiotic powder containing the same strains as the intervention group	12 weeks	Double-blind RCT (*N* = 40)	N/A	Active, Not recruiting Estimated completion date Dec 2023	NCT05145881
Probiotic supplement containing: - *Lactobacillus acidophilus* - *Lactobacillus casei* - *Bifidobacterium bifidum* - *Lactobacillus fermentum*	AD	Placebo	12 weeks, 24 weeks	Observational study (*N* = 240)	N/A	Recruiting Estimated completion date Feb 2025	NCT05943925
Probiotic powder containing: - *Bifidobacterium animalis subsp. lactis* BS01 - *Bifidobacterium longum* BL03 - *Bifidobacterium adolescentis* BA02 - Fructo-oligosaccharides - Maltodextrin	PD	Placebo powder (Maltodextrin)	12 weeks	Double-blind, placebo-controlled RCT (*N* = 88)	N/A	Enrolling by invitation Estimated completion date Jan 2023	NCT05173701
Probiotic powder (Ecologic® BARRIER 849) containing: - *Bifidobacterium bifidum* - *Bifidobacterium* lactis - *Lactobacillus acidophilus* - *Lactobacillus brevis* - *Lactobacillus casei* - *Lactobacillus salivarius* - *Lactococcus lactis*	PD	Placebo powder	12 weeks	Triple-blind, placebo-controlled RCT (*N* = 60)	Phase II	Not yet recruiting Estimated completion date Oct 2026	NCT05568498
Probiotic powder (Ecologic® BARRIER 849) containing: - *Bifidobacterium bifidum* - *Bifidobacterium* lactis - *Lactobacillus acidophilus* - *Lactobacillus brevis* - *Lactobacillus casei* - *Lactobacillus salivarius* - *Lactococcus lactis*	PD	Placebo powder	12 weeks	Triple-blind, placebo-controlled RCT (*N* = 61)	Phase II	Active, Not recruiting Estimated completion date Dec 2023	NCT03968133
Bifidobacterium triple viable capsules (BIFICO) containing: - *Bifidobacterium longum* - *Lactobacillus acidophilus* - *Enterococcus faecalis*	PD	Placebo capsules	24 weeks	Double-blind, placebo-controlled RCT (*N* = 240)	Phase IV	Recruiting Estimated completion date Dec 2023	NCT04871464
Probiotic capsules containing: - *Lactobacillus Plantarum* PS128	PD	Placebo capsules	12 weeks	Double-blind, placebo-controlled RCT (*N* = 120)	N/A	Unknown Status Estimated completion date Jun 2023	NCT04722211
Probiotic sachets (Enterolactis duo®) containing: - *Lactobacillus casei*	PD	N/A	12 weeks	Open-label, single-group assignment (*N* = 30)	N/A	Recruiting Estimated completion date Jun 2023	NCT04293159
Multi-strain liquid probiotic (Symprove) containing: - *Lactobacillus rhamnosus* - *Enterococcus faecium* - *Lactobacillus acidophilus* - *Lactobacillus plantarum*	PD	Placebo	12 weeks	Double-blind, placebo-controlled RCT (*N* = 60)	N/A	Active, Not recruiting Estimated completion date Jul 2023	NCT05146921
Proprietary probiotic formulation	ALS	N/A	6 months	Open-label, non-randomized two-group pilot study (*N* = 5)	N/A	Completion date Jun 2019 No results posted	NCT03324399
Synbiotics (probiotic + prebiotic)							
Standard dopamine replacement therapy with or without prebiotic fibers and probiotic *Lactobacillus acidophilus*	PD	Standard dopamine replacement therapy	3 months	Open-label RCT (*N* = 66)	Phase III	Recruiting Estimated completion date Jun 2025	NCT05576818
Fecal microbiota transplantation							
FMT from a healthy donor via colonoscopy Pre-treatment with antibiotic rifaximin for 7 days	PD	Autologous FMT	12 months	RCT (*N* = 120)	Phase I & II	Enrolling by invitation Estimated completion date Dec 2024	NCT05204641
Intracecal FMT from a healthy donor	PD	Intracecal infusion of placebo solution (sodium chloride + glycerol mixture)	6 months	Double-blind, placebo-controlled RCT (*N* = 51)	N/A	Active, Not recruiting Estimated completion date Jun 2023	NCT04854291
Nasojejunal FMT from a healthy donor	PD	Autologous FMT	12 months	Double-blind, placebo-controlled RCT (*N* = 49)	N/A	Completion date Dec 2022 No results posted	NCT03808389
FMT (route and source not stated)	PD	N/A	6 months	Open-label, single-group assignment (*N* = 30)	N/A	Recruiting Estimated completion date Dec 2025	NCT04837313
FMT from a healthy donor via duodenum-jejunum infusion	ALS	No procedure	12 months	Double-blind, placebo-controlled RCT (*N* = 42)	N/A	Active, Not recruiting Estimated completion date Aug 2024	NCT03766321

Despite advancements in understanding gut–brain communications and encouraging results in experimental models, the clinical trials of microbiome-based therapeutics have yielded mixed results. Microbiome-based therapeutics generally exhibit favorable safety and tolerability profiles, alongside some degree of clinical efficacy in improving cognitive function and motor function. Moreover, these interventions are effective in improving constipation-related parameters in PD patients, including bowel movements, gut transit time, stool consistency, and quality of life related to constipation. However, a notable limitation of the current studies is the lack of gut microbiome profiling, which hinders the comparison of taxonomic composition and metabolomic profiles before and after the intervention. In addition, there is a paucity of clinical trials exploring microbiome-based therapeutics in ALS, FTD, and HD.

The application of probiotics and prebiotics is limited by several factors. A notable limitation of probiotics is their suboptimal therapeutic efficacy, primarily attributed to their vulnerability to low gastric pH and exposure to various digestive enzymes, resulting in probiotic inactivation and impaired bioactivity. In addition, the low mucoadhesive capability and insufficient intestinal retention of probiotics greatly hamper their intestinal colonization.^733^ Several innovative strategies are currently being investigated to enhance the oral bioavailability and intestinal targeting capability of probiotics. These include microencapsulation,^734^ hydrogel encapsulation,^735^ nanoparticle encapsulation,^733^ integration with nanozyme,^736^ nanocoating,^737^ mineral coating,^738^ and optogenetic probiotic system.^739,740^

On the other hand, it is difficult to predict and control the outcomes of microbiome manipulation by prebiotics, as they affect the growth and activities of multiple microbial species.^728,741^ Nevertheless, ongoing efforts are focused on understanding how dietary fibers with different structures modulate the human gut microbiome.^742^ Furthermore, growing studies are exploring the strategy of precision manipulation of the gut microbiota to achieve specific health endpoints.^742–745^ Moreover, the effectiveness of prebiotics varies depending on the native microbiota. Prebiotics are more likely to be effective when the necessary bacterial strains are already present in the gut. However, these beneficial strains may be lacking in certain medical conditions. Thus, it is essential to administer the missing symbiotic bacterial strains to restore and support a healthy gut microbiota.^728^ A refined understanding of the mechanisms governing the metabolism and absorption of prebiotics will inform the rational design of clinical trials.

Another notable trend is the limited number of trials investigating the applications of FMT in the treatment of neurodegenerative diseases. An evident gap in our understanding pertains to the “ideal” gut microbiome that resists neurodegeneration, thereby significantly complicating the identification of the most suitable donors. Similar limitations are common in the application of FMT in anticancer treatment. Interestingly, increasing studies are investigating the use of fecal samples from cancer patients who exhibited complete and durable responses to immune checkpoint inhibitors.^726^ This approach holds promise for investigation in the context of neurodegenerative diseases.

In addition, FMT is limited by its variable degree of engraftment, limited scalability, and a lack of standardized protocols.^725,726^ The extent of microbial engraftment is an important proxy of the clinical success of FMT.^746,747^ To improve FMT engraftment, antibiotic preconditioning for the recipient has been recommended, based on two recent meta-analyses that reported a higher degree of donor microbiome engraftment in recipients with a precarious microbial community.^746–748^ Moreover, combined routes of FMT administration are an effective strategy to enhance strain engraftment.^747^ Another point of consideration is the necessity of repeated FMT administration to counteract the persistent disease-driving forces that affect the gut ecological niche and achieve long-term efficacy. This clinical requirement underscores the critical need for a standardized FMT product with excellent stability and reproducibility.^746^ FMT may also carry the risk of unintended introduction of pathogens and antibiotic-resistant microbes into the recipients.^749,750^ Thus, it is imperative to screen the donor samples prior to the FMT to mitigate these risks.^728^

## Conclusions

The unabated rise in the global prevalence of neurodegenerative diseases,^751^ coupled with the suboptimal therapeutic outcomes of current FDA-approved drugs, emphasize the need for an alternative strategy to discover effective therapeutic targets. The microbiota–gut–brain axis represents an important regulator of glial functions, making it an actionable target for ameliorating the development and progression of neurodegenerative diseases. With the aid of cutting-edge technologies, we are beginning to delineate the intricate communication between gut microbiota and glial cells in neurodegenerative diseases. A dysregulated gut microbiome adversely affects glial cells by compromising the integrity of the intestinal barrier and BBB, with recent evidence also revealing the involvement of the meningeal barrier. The available preclinical evidence supports the use of probiotics, prebiotics, and FMT to attenuate glial activation and cognitive impairment by restoring the integrity of the intestinal barrier and BBB. Nevertheless, the clinical translation of microbiome-based therapeutics remains challenging, underscoring the need for continued research efforts to unravel the complexities of the microbiota–gut–brain axis and fully harness their potential.

Establishing a definitive causal relationship between the altered gut microbiome and disease remains challenging as it is difficult to determine whether the observed microbiome alterations are causative, consequential, or merely a bystander response to the disease. Moreover, existing animal models do not fully recapitulate the intricacies of the human microbiome and pathobiology. Thus, the excessively high rate (95%) of positive results and causal claims in human microbiota-associated rodents demands caution against overinterpreting and overstating the causal implications of these findings.^752^ Nevertheless, animal models, when combined with single-cell technologies and computational techniques, remain essential complementary tools as they offer valuable mechanistic insights that are difficult to obtain through human studies.

The increasing maturity of technical and methodological innovations has enabled us to unravel numerous aspects of the microbiota–gut–brain axis and discover opportunities for therapeutic development. Notably, the recent development of non-invasive, ingestible sampling devices has made it possible to collect luminal contents throughout the intestinal tract, potentially overcoming the limitations of stool samples in reflecting the regional variation of gut microbiota.^354,753^ In addition, we are beginning to understand the intricate cell-to-cell signaling mechanisms of gut microbiota that regulate community behaviors.^754^ Ultimately, the malleability of the human gut microbiome presents exciting opportunities for the development of personalized microbiome-based therapeutics for neurodegenerative diseases.
